# Origin of the Anomalous Guest Polymer Selectivity in Complex Coacervates

**DOI:** 10.1021/acsnano.6c06413

**Published:** 2026-09-29

**Authors:** Zongpei Wu, Júlia Bonesso Sabadini, Pierre J. Walker, Watson Loh, Zhen-Gang Wang, Sarah L. Perry, Shensheng Chen

**Affiliations:** † Department of Chemical and Biological Engineering, 58207 The Hong Kong University of Science and Technology, Clear Water Bay, Hong Kong 999077, China; ‡ Institute of Chemistry, 344102 University of Campinas (UNICAMP), P.O. Box 6154, 13083-970 Campinas, São Paulo, Brazil; § Division of Chemistry and Chemical Engineering, 6469 California Institute of Technology, Pasadena, California 91125, United States; ∥ Department of Chemical and Biomolecular Engineering, 28132 University of Massachusetts Amherst, Amherst, Massachusetts 01003, United States

**Keywords:** polyelectrolyte complex coacervates, guest encapsulation, guest polymer interaction, electrostatic correlations, electrostatic exclusion, liquid−liquid phase separation

## Abstract

Complex coacervates—liquid condensates resulting from liquid–liquid phase separation of oppositely charged polymers—have attracted broad interest in artificial cells, drug delivery vehicles, and smart nanoreactors. The functionality of these applications is significantly influenced by the coacervate’s ability to encapsulate guest macromolecules, where the guest interactions determine their loading efficiency and activity. Using MD simulations, analytical theory, and experiments, we demonstrate that the intrinsic electrostatic correlations within the coacervate matrix significantly modify the effective interactions of guest polymers in the dense phase, resulting in the strong dependence of encapsulation on guest chain length, charge density, and charge sequence observed experimentally. Even in the absence of chemical incompatibility, neutral polymers are strongly excluded from the coacervate phase as a result of disrupting electrostatic correlations within coacervates. Consequently, the encapsulation efficiency of neutral polymers exponentially decays with increasing guest chain length. For polyampholyte-like intrinsically disordered proteins (IDPs), their effective interaction within coacervates is largely determined by charge sequence: IDPs with more-disordered sequences experience strict rejection from the dense phase, whereas those with more blocky charges are dispersed within coacervates. Interestingly, for all the guest species tested, their effective interactions are opposite to those observed in dilute solutions, where the sign of the effective two-body interaction reverses when going from the dilute phase to the dense coacervate phase. We elucidate that the non-trivial guest selectivity in complex coacervates originates from the disruption of polycation–polyanion electrostatic correlations by the guest, which combines an energetic penalty (dilution of charge density) with an entropic penalty (loss of chain conformational freedom of the host). These findings have significant implications for understanding guest behavior in coacervate and biocondensate systems.

## Graphical abstract

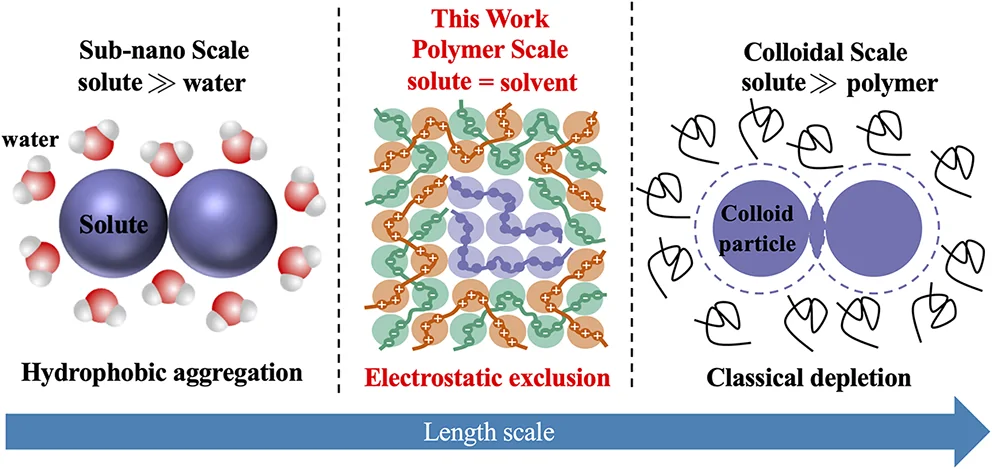

## Introduction

Life relies on the intricate functions of biomacromolecules operating within the crowded environments of living cells. Complex coacervates—macromolecular condensates resulting from electrostatic associations of oppositely charged polymers such as proteins and RNA—are increasingly recognized as crucial media for intracellular activities.^1–4^ These charge-rich, liquid-like coacervates create microenvironments that selectively partition biomacromolecules and drive biochemical reactions, thereby regulating cellular organization and biochemistry. Inspired by their unique ability to encapsulate functional polymers, proteins, and enzymes in aqueous environments, researchers are exploring coacervate-based systems for innovative applications in artificial cells,^5–7^ biomedicines,^8,9^ and functional nanoreactors.^10,11^ Central to these functions is the interaction of guest polymers within the coacervate matrix, which ultimately governs their thermodynamic stability and local concentration. However, despite significant advances in characterizing coacervates themselves,^12,13^ the fundamental principles governing guest polymer behavior within these densely charged coacervate environments remain poorly understood, leaving a critical gap in our ability to predict and control macromolecule sequestration.

A particularly intriguing puzzle is the origin of the strict encapsulation selectivity on guest polymers observed in typical complex coacervates. While highly charged complex coacervates can enrich many types of small guest molecules such as organic dyes,^14,15^ small molecule metabolites,^16,17^ and small proteins,^8,18,19^ they often strictly exclude macromolecules even when those polymers have a similar chemical identity to the encapsulated small molecules. For example, while both small sugar (sucrose) and poly­(ethylene glycol) (PEG) are neutral and hydrophilic, complex coacervates can encapsulate sucrose but tend to exclude larger PEGs.^20,21^ A similar disparity exists for dextran: whereas small dextran (4 kDa) is rapidly sequestered and concentrated within the PDDA/ATP coacervate, large dextran (70 kDa to 500 kDa) is entirely excluded from the dense phase.^22^ Since both PEG and dextran are neutral, hydrophilic, and contain monomers with seemingly favorable interactions with the coacervate matrix, their ubiquitous exclusion from highly charged coacervates cannot be explained by the unfavorable interaction within the traditional Flory–Huggins framework.

Besides neutral guest polymers, complex coacervates also exhibit non-trivial encapsulation selectivity on charged guest polymers, including polyelectrolytes and proteins. Intuitively, coacervates should favor charged guest polyelectrolyte/proteins which contribute to the macromolecular charge neutrality in the coacervate matrix. However, Obermeyer et al.,^23–25^ report that coacervates can absorb significant amount of “supercharged” guest proteins with high-density charge patches, even if encapsulating those proteins will substantially break macromolecular charge neutrality in the coacervates. In contrast, guest proteins with disordered charge distributions usually result in much lower encapsulation efficiency compared to their charged-patchy counterparts.^23,26^ These observations suggest that sequestration of charged polymers in coacervates is governed by mechanisms much more complicated than macromolecular charge compensation. This non-trivial selectivity on both neutral and charged guest polymers seems to be a unique signature of coacervates, as this does not occur in simple liquids or simple polymer solutions. Unlike classical micellar solubilization, where guest uptake is driven by hydrophobic mismatch with the bulk solvent, the underlying driving force of guest encapsulation in coacervate is more strongly driven by electrostatic interactions. The strong sensitivity of coacervates to the chain length and charge sequences of the guest polymers indicates that the intrinsic microstructure of coacervates gives rise to substantial modifications of the effective interactions between guest polymers within the coacervate matrix. The most unique characteristic of complex coacervates is the electrostatic correlation between oppositely charged polycations and polyanions.

Voorn and Overbeek^27^ first demonstrated that the positive–negative electrostatic correlations between charged macromolecules are responsible for the formation of coacervates. The original Voorn–Overbeek framework has been further improved by Olvera de la Cruz,^28,29^ de Pablo,^30–32^ Wang,^33–36^ Fredrickson,^37,38^ Qin,^31,39^ Larson,^40,41^ Erukhimovich,^42^ and Sing^43,44^ to account for the effects of excluded volume interactions, chain connectivity, charge distributions, solvent contribution, pH, etc., as summarized in two recent reviews.^12,13^ Recent scattering experiments and theory have further confirmed the charge correlations between oppositely charged species within the coacervate matrix.^45^ These advancements highlight the importance of electrostatic correlations in the coacervate matrix, underscoring their role in driving complex coacervation and regulating salt partitioning. However, the role of electrostatic correlations within the coacervate matrix on the behavior of guest polymers, on the other hand, remains largely unexplored. Using Random Phase Approximation (RPA) to model complex coacervation, Chen et al.^46,47^ showed that the mismatched charge density between a third polyelectrolyte can lead to the coexistence of two charge-dense phases with a dilute phase, driven purely by electrostatic correlations. The formation of a third phase can be taken to indicate the expulsion of the third “guest” polyelectrolyte from the coacervate. At the microscopic level, when guest chains represent the minority component, it remains unclear how electrostatic correlations within coacervates impact the interactions between guests, and how this then relates to the non-trivial guest-polymer selectivity observed in experiments.

In this study, using extensive molecular dynamics (MD) simulations, analytical theory, and supporting experiments, we show that strong polycation-polyanion electrostatic correlations within complex coacervates significantly modify the effective interaction of various guest polymers, resulting in non-trivial guest encapsulation efficiency. Computationally, by examining the potential of mean force (PMF) between two nanoscale guest polymers within the coacervate, we show that the interaction between two neutral guest chains will change from repulsive to attractive once their chain length exceeds a few nanometers, due to the disruption of local electrostatic correlations of the host coacervate matrix. The guest polyelectrolytes and guest disordered proteins show similar and richer behavior, highlighting an “electrostatic exclusion” force possessed by the coacervate matrix. In our simulated systems, we focus on moderately short chains where guest and host polymers are of similar size, with the radii of gyration falling into the range of 0.9 nm–2.6 nm (see Figure S6 in the Supporting Materials).

## Results and Discussion

### Strong Guest Aggregation Tendency within Complex Coacervates

The complex coacervates in our simulations result from liquid–liquid phase separation of symmetric, oppositely-charged polyelectrolyte solutions (see Figure S1 in Supporting Materials), representing generic electrostatically-driven coacervate systems in both synthetic and biological contexts (Figure 1A). We then introduce approximately 1% guest polymers (w/w) to the coacervate matrix and monitor their behavior. To focus on electrostatic effects, we set the excluded volume interactions to be the same among all species (polycations, polyanions, water, counterions, and guest polymers), representing good solvent conditions for all polymers, i.e., χ = 0 among all species with χ being the Flory–Huggins parameter. For the majority of our simulations, we focus on guest polymer behavior within the coacervate phase without simulating the coexisting supernatant phase. As such, guest aggregation within the coacervate (disfavored by the host) means that guests will be expelled into the supernatant phase or form a separate phase.^46^

**Figure 1:**
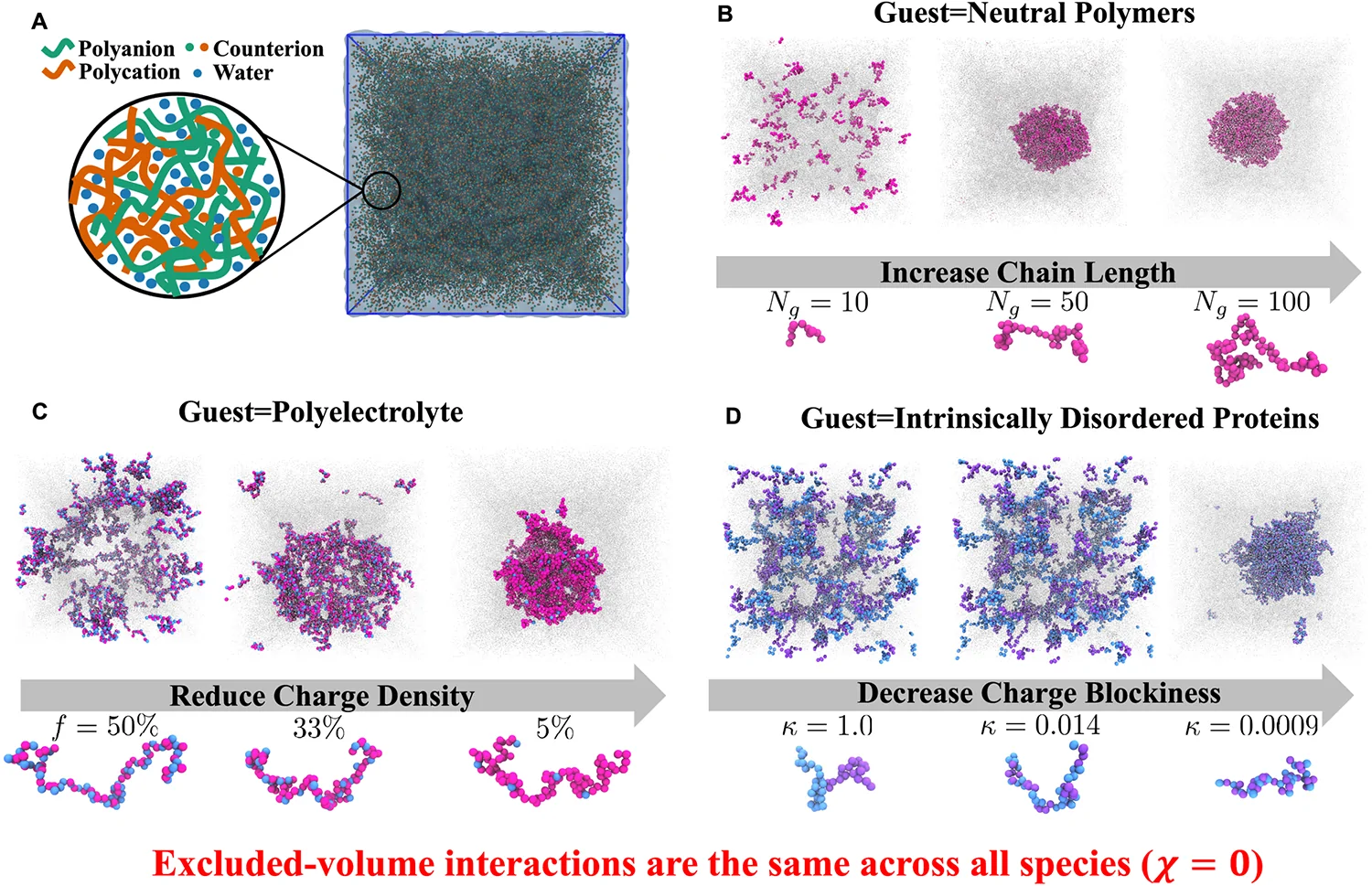
The behavior of guest neutral polymers, polyelectrolytes, and polyampholyte-like IDPs within the coacervate phase (without a coexisting supernatant), where χ = 0 across all species. (A) The composition of a typical coacervate system (left: schematic; right: simulation snapshot). (B) Aggregation/dispersion states of 1% guest neutral polymer with chain length of *N*_*g*_ = 10, 50, 100. (C) Guest polyelectrolytes of different charge densities (*f* = 5%, 33%, 50%) within the coacervate matrix. (D) The behavior of fully-charged IDPs with different charge sequences (κ = 0.0009, 0.014, 1.0) in the coacervate matrix. Green, orange, and blue (transparent) beads in A represent polycation monomers, polyanion monomers, and water beads, respectively. In (B–D), gray beads represent monomers of the background coacervate matrix; magenta, blue, and purple represent the neutral, negatively charged, and positively charged monomers of the guests, respectively. The excluded volume interactions are the same across all species.

Figure 1 shows that, even in the absence of chemical incompatibility, most guest polymers tend to form aggregates within the coacervate matrix, i.e., they have unfavorable interactions with the coacervate matrix and will be excluded from the coacervate phase. The aggregation tendency is particularly pronounced for neutral polymers: when the guest chain length is above some critical chain length (*N*_*g*_ ≳ 30), all neutral chains will immediately aggregate into a globule within the coacervate (Figure 1B), while only very short chains (e.g., *N*_*g*_ = 10) remain relatively dispersed (left panel in Figure 1B). The aggregation behavior of neutral guest polymers seems to be independent of the chain length of host polyelectrolytes (*N*_*h*_), as the guest polymers (*N*_*g*_ = 50) show qualitatively similar behavior for both *N*_*g*_ > *N*_*h*_ = 30 (Figure 1B) and *N*_*g*_ < *N*_*h*_ = 100 (Figure S2 in the Supporting Materials). At the single-chain level, we find that the guest chain becomes more compressed relative to its ideal chain state as chain length increases (Figure S6 in the Supporting Materials), again highlighting that coacervates make the guest chains more attractive. The underlying mechanism for such chain aggregation is conceptually similar to the “depletion-mediated” polymer collapse observed in particle-polymer systems.^48^ However, as both our guest and host are flexible chains of similar size, the origin of guest aggregation is not the same as the traditional depletion effect^49,50^ , but more likely relates to electrostatic interactions.

The ubiquitous guest exclusion also occurs for charged guests, including low-charge-density polyelectrolytes and fully-charged polyampholyte-like intrinsically disordered proteins (IDPs) with an overall neutral charge. For guest polyelectrolytes, we find that exclusion from the coacervate phase—manifested by their aggregation within coacervates—can be weakened by increasing charge density (Figure 1C). For instance, at a low charge density of *f* = 5%, all guest polyelectrolytes experience strong exclusion from the coacervate phase and aggregate into a compact globule, similar to the case for the neutral guest polymers. The aggregation tendency persists until their charge density increases to *f* ≈ 50% (where one in every two monomers carries +1*e* charge, Figure 1C). For polyampholyte-like IDPs, their aggregation/dispersion behavior within coacervates are highly dependent on their charge sequence, characterized by the parameter *κ*, where a smaller *κ* indicates the charge distribution is more disordered, and vice versa^51^ (see Section S2 in Supporting Materials). As illustrated in Figure 1D, IDPs with more disordered sequences tend to experience stronger exclusion and rapidly aggregate within the coacervate phase, while diblock IDPs (κ = 1.0) remain dispersed (Figure 1D). In real IDPs, charges are more sparse (lower charged density), and large blocks of like charge are less common. Therefore, we expect more prevalent IDP aggregation within coacervates. Indeed, if IDP charge density is reduced to *f* = 50%, we find the critical charge blockiness is κ^*c*^ ≈ 0.14, below which the IDPs remain aggregated (see Figure S4 in Supporting Materials).

The strong tendency for guest polymers to aggregate within the coacervate, even when they are partially charged (polyelectrolytes) or carry both positive and negative charges (IDPs), indicates an inherent, strong driving force in the coacervate that effectively denies guests. Below, we compute the effective interaction (free energy) between two guest chains within the coacervate phase.

### Effective Guest–Guest Interaction within Coacervates

To quantitatively measure the effective interactions of guests, we calculate the potential-of-mean-force (PMF) Δ*F*(*r*) between two guest polymers as a function of their center-of-mass distance Δ*r* within the coacervate phase. For comparison, we also measure their PMFs in the supernatant phase that coexists with the coacervate. Within the coacervate, we find that neutral guest polymers with longer chain lengths *N*_*g*_ experience stronger effective attraction, evidenced by the deeper and wider free energy wells (Figure 2B). While very short guest chains (*N*_*g*_ = 10) exhibit weak repulsion, longer chains become increasingly attractive, with the PMF minimum dropping from −1.0*k*_B_ *T* for *N*_*g*_ = 50 to −4.0*k*_B_ *T* for *N*_*g*_ = 100, where *k*_B_ is the Boltzmann constant, and *T* is the temperature. Consequently, as we gradually increase the guest chain length (*N*_*g*_ = 10, 16, 30, 50, 100), we observe that guest chains rapidly aggregate into clusters for *N*_*g*_ ≥ 30, as manifested by the progressively long sub-diffusive regime in the mean-squared-displacement ⟨*r* ^2^(Δ*t*)⟩ (Figure 2D). The interaction strength between the two neutral guest polymers also increases with the chain length of the host polyelectrolytes. For a guest polymer with chain length *N*_*g*_ = 50, their PMF minimum deepens from −1.0*k*_B_ *T* to −2.2*k*_B_ *T* when the host chain length increases from *N*_*h*_ = 30 to *N*_*h*_ = 100 (Figure 2C). The effective two-chain attraction gives rise to the universal aggregation of the guest chains, as shown in Figure 1B. We find that at the repulsive-attractive transition, the chain length of the guest chains (*N*_*g*_ = 30) corresponds to a radius of gyration of around 1.6 nm, indicating that the nature of guest interactions can be altered by nanoscale mechanisms.

**Figure 2:**
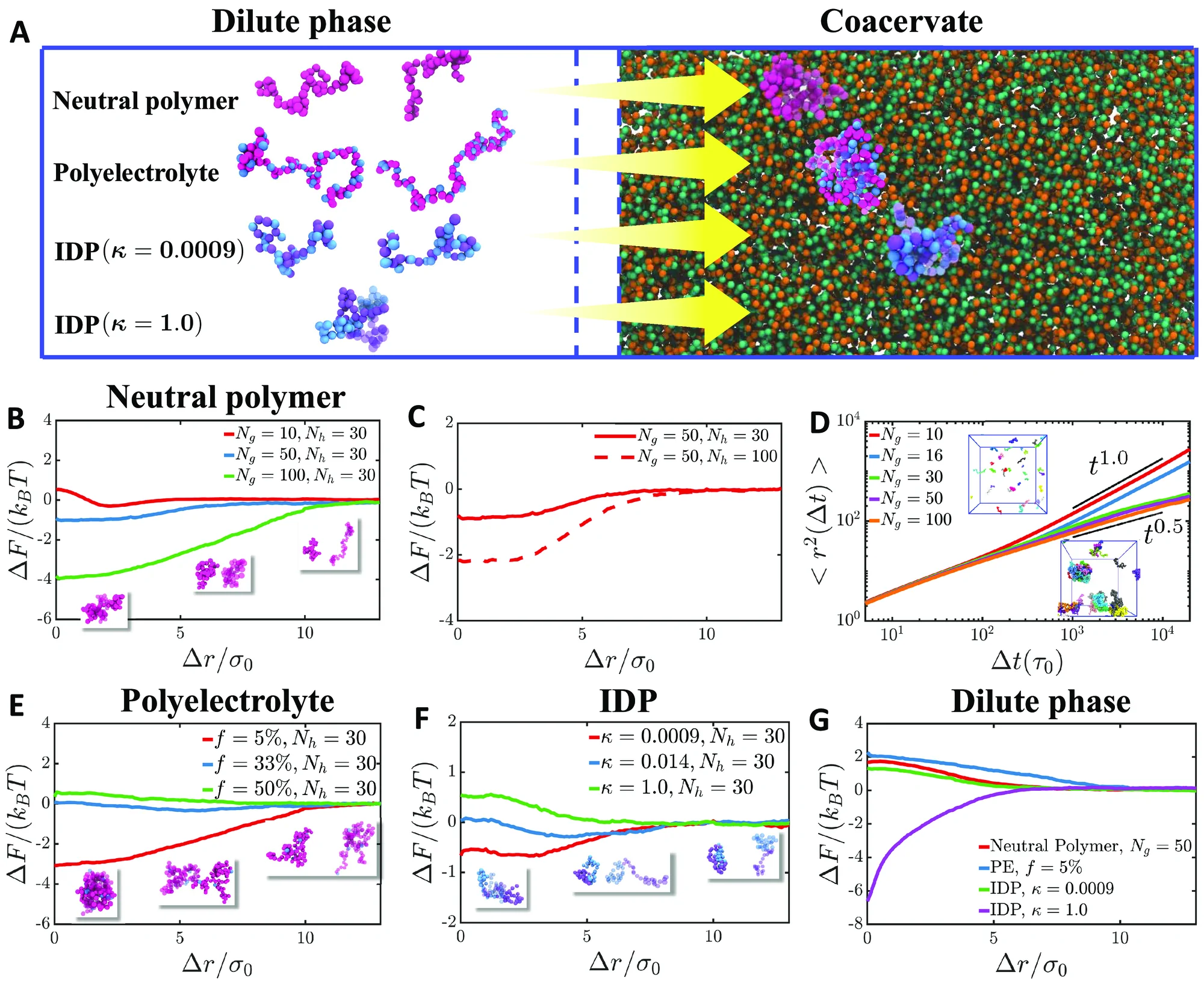
Effective guest–guest interaction within the coacervate phase. (A) Schematic comparisons of guest behavior within the dilute phase (left) and coacervate (right). (B) Potential-of-mean-force (PMF) between two neutral guest polymers in coacervate (*N*_*h*_ = 30) with different guest chain length of *N*_*g*_ = 10, 50, 100. (C) PMFs of two neutral guest (*N*_*g*_ = 50) in coacervates with different host chain length *N*_*h*_ = 30 and *N*_*h*_ = 100. (D) Mean-squared-displacement (MSD) of neutral guest polymers for different *N*_*g*_. The upper and lower insets are representative snapshots for systems with *N*_*g*_ = 10 and *N*_*g*_ = 100, respectively, where *N*_*g*_ = 100 shows clustering of guest chains and exhibit substantial sub-diffusion. (E) PMFs of guest polyelectrolytes at charged density *f* = 5%, 33%, and 50% in coacervate with *N*_*h*_ = 30. (F) PMFs of fully charged IDPs with different charge sequences at κ = 0.0009, κ = 0.014, and κ = 1.0. (G) PMFs of guest polymers at dilute phase. Magenta, blue, and purple beads represent the neutral, negatively charged, and positively charged monomers of guest polymers, respectively. The green and orange beads represent the polycations and polyanions in the host coacervates. The insets in the PMF plot are the simulation snapshots at representative separation distances Δ*r*.

The interaction between two low-charge-density (*f* = 5%) guest polyelectrolytes (PEs) also shows an attractive PMF, with a free energy minimum of about −3*k*_B_ *T*, comparable to that of neutral polymers (Figure 2E). Notably, the inter-chain attraction persists even as the charge density increases to *f* = 33%, and only diminishes until *f* = 50%. The two-body attraction between guest polyelectrolytes provides a microscopic rationale for recent findings on the formation of an additional charge-dense phase when coacervate components have strong mismatch in charge density.^46,52^ The attractive PMFs also persist for interactions between fully charged IDPs with disordered sequences (κ = 0.014), driving IDP aggregation within coacervates (Figure 2A,2F). For example, IDPs with κ = 0.0009 show a free energy well with a depth of −0.7*k*_B_ *T* (Figure 2F), which diminishes as charge blockiness increases from κ = 0.0009 to κ = 0.014, and eventually disappears when κ > 0.014. When the κ increases to 1.0, the inter-chain interaction becomes repulsive (Figure 2F), resulting in dispersed IDPs within the coacervate phase (Figure 2A).

Interestingly, guest polymers exhibit drastically different behavior in the coacervate phase compared to the dilute phase, where the sign of the effective two-body interaction reverses when going from the dilute phase to the coacervate (Figure 2A). In good solvent conditions (as in this work), the PMF for both neutral polymers and low-charge-density polyelectrolytes is repulsive in the dilute phase (Figure 2G), leading to dispersion (left panel in Figures 2A and S3 in Supporting Materials). In contrast, both types aggregate as a compact globule in the coacervate phase. This reversal also applies to IDPs: in the dilute phase, it is known that IDPs with high κ, such as κ = 1.0, will undergo self-coacervation^51,53,54^ to form aggregates, as evidenced by their attractive PMF (Figure 2G). Conversely, these IDPs become repulsive within the coacervate phase (Figure 2A,2F). On the other hand, low-κ IDPs are repulsive within the dilute phase but become attractive in the coacervate phase (Figure 2F,G). Evidently, the densely charged, crowded coacervate environment significantly alters both the structure and thermodynamics of guest macromolecules. Notably, the partition preference of guest molecules is strictly determined by the single-guest transfer free energy rather than by the guest–guest pair PMF directly. Here, we use the guest–guest PMF as a proxy for guest exclusion within the coacervate, and the phase diagrams in Figure 4 address the partitioning behavior directly.

### Experimental Evidence of Strong Guest Aggregation Force

Direct experimental comparison with simulations is challenging due to the difficulty of identifying polymer systems (polycation, polyanion, and guest) with a vanishing Flory–Huggins interaction parameter (χ) across all species, as well as the high material requirements for preparing macroscopic coacervates for encapsulation. To experimentally highlight this strong aggregation force, we mixed a weakly associated “guest” coacervate with a strongly associated “mother” coacervate, where the “guest” phase comprises the guest polymer and one of the polyelectrolyte species present in the “mother” phase. To achieve this, we formulated systems comprising three distinct charged species: the polyanion *E*_48_ and a pair of polycations selected from poly­(allylamine) (PAH), poly­(lysine) with *N* = 48 (*K*_48_), and (KG)_24_ (a polypeptide consisting of 24 alternating positively charged lysine and neutral glycine residues). This strategy allows us to systematically evaluate the influence of polycation charge density on the resulting segregation from the “mother” coacervate.

To test this, both the low-charge-density polypeptide (KG)_24_ and the higher-charge-density *K*_48_ were individually mixed with PAH and allowed to complex with the polyanion *E*_48_. Isothermal titration calorimetry (ITC) measurements of the individually complexing species demonstrated that PAH forms a significantly stronger complex with *E*_48_ (binding free energy Δ*G*° = −*RT* ln­(*K*_a_) ≈ −7.9 kcal/mol, where *R* is the universal gas constant and *K*_*a*_ is the binding constant) than either *K*_48_ (Δ*G*° ≈ −6.5 kcal/mol) or (KG)_24_ (Δ*G*° ≈ −6.2 kcal/mol). Despite the notable energetic attraction between these cationic polypeptides and the host polyanion, both *K*_48_ and (KG)_24_ are excluded from the strongly associated PAH-rich inner coacervate phase, resulting in multiphase compartmentalization (Figure 3A,C). In contrast, guest polymers remain fully dispersed within the mother coacervate (forming a macroscopic single-phase system) only when their association strength with the oppositely charged host is comparable to the association strength governing the mother coacervate itself. For instance, because the binding affinities of *K*_48_-*E*_48_ and (KG)_24_-*E*_48_ are nearly equivalent (Figure 3D), mixing these complexes yields a homogeneous, single-phase droplet with fully co-localized polycations (Figure 3B).

**Figure 3:**
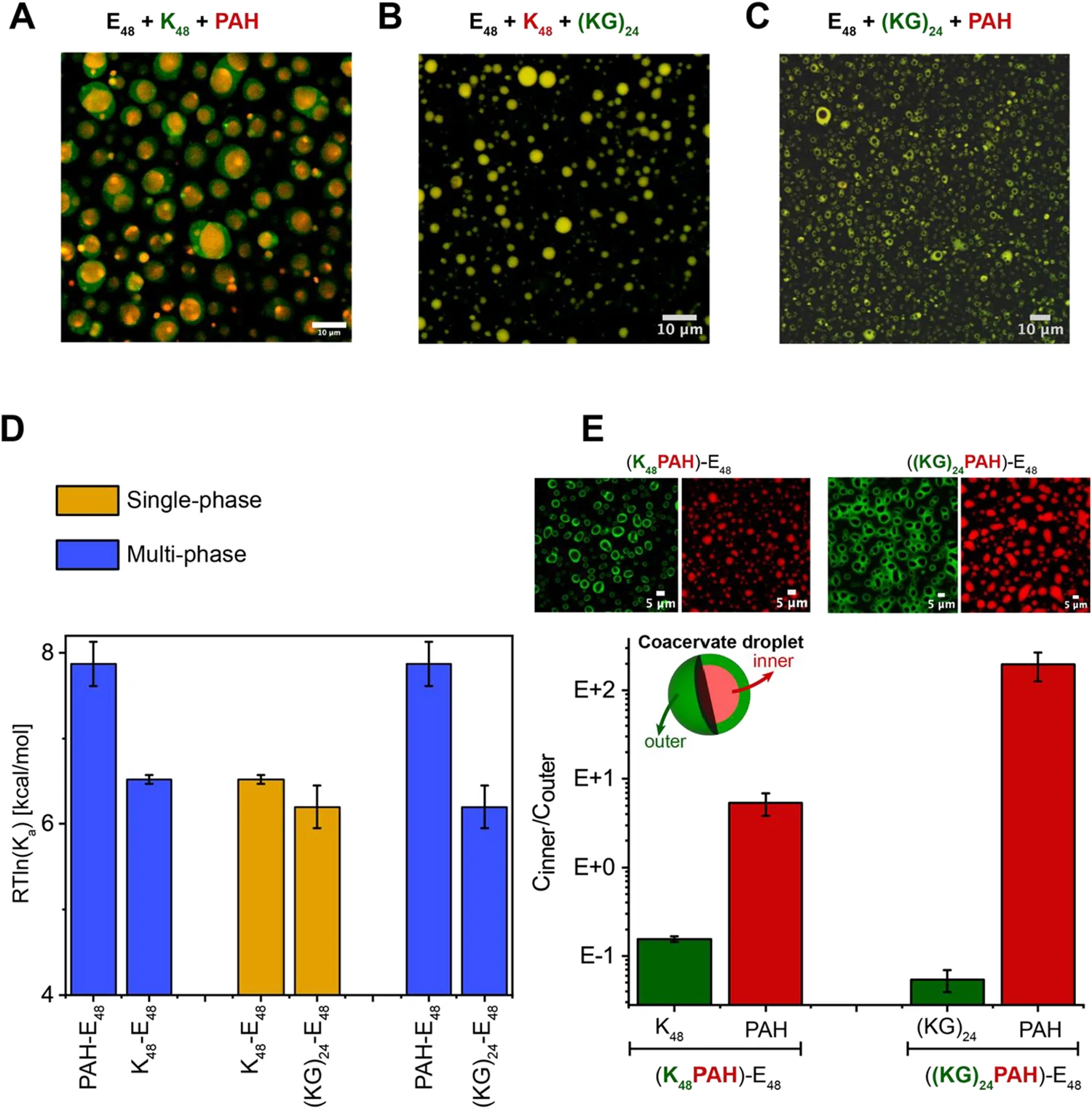
Phase morphology, binding thermodynamics, and spatial partitioning of multicomponent complex coacervates. (A–C) Confocal fluorescence micrographs of coacervates prepared by mixing the polyanion *E*_48_ with distinct pairs of polycations. (A) *E*_48_ mixed with *K*_48_ (green) and PAH (red) forms a compartmentalized multiphase system. (B) *E*_48_ mixed with *K*_48_ (red) and (KG)_24_ (green) forms a homogeneous single-phase system, resulting in co-localization (yellow). (C) *E*_48_ mixed with (KG)_24_ (green) and PAH (red) forms a multiphase system. (D) Pairwise binding constants (*RT* ln­(*K*_*a*_)) for each polycation-*E*_48_ complex obtained using isothermal titration calorimetry (ITC). Bar colors designate whether mixing the corresponding binary coacervates yields a macroscopic single-phase (yellow) or multi-phase (blue) state. (E) Quantitative analysis of polycation partitioning within the multiphase coacervate systems. The top panels display isolated fluorescence species to visualize the spatial segregation of the polycations within the droplets. The bar chart quantifies the concentration ratio of each polycation between the inner and outer coacervate phases (*C*_inne*r*_/*C*_outer_). In both multiphase systems, PAH strongly segregates into the inner dispersed phase, whereas *K*_48_ and (KG)_24_ predominantly partition into the outer continuous phase. Error bars indicate standard deviation.

In agreement with our computational predictions, this electrostatic exclusion is amplified as the guest charge density decreases. As quantified by the inner-to-outer concentration ratios (*C*_inner_/*C*_outer_) in Figure 3E, the lower-charge-density (KG)_24_ exhibits a more pronounced segregation from the PAH mother phase compared to *K*_48_. These results corroborate the simulation findings, demonstrating that low-charge-density polyelectrolytes experience more significant repulsion from the dense mother phase, driving them to aggregate into a distinct segregated domain.

### Physical Origin of the Exclusion of Guest Polymers

The exclusion of guest polymers from coacervates originates from the electrostatic correlations that stabilize the complex coacervate. When guest polymers are introduced, they perturb these correlations; if the disruption is sufficiently large, the guest is expelled from the coacervate. Although the underlying principle is universal—guest-induced disruption of electrostatic correlations—the detailed mechanism depends on the characteristics of the guest polymer. To elucidate these differences, we employ liquid-state^55,56^ (LS) theory, which we have previously used to accurately model the phase behavior of host polyelectrolyte coacervates.^57^ Within the bounds of a mean-field approximation, LS theory accounts for polymer connectivity and long-range electrostatics, both of which are essential for describing complex coacervate systems.

LS theory provides an explicit expression for the free energy of an incompressible mixture of equal-sized beads

1 $$F=F_{\mathrm{id}}+F_{\mathrm{el}}+F_{\mathrm{ch}}$$

where *F*_id_ is the translational (Flory–Huggins) entropy, *F*_el_ captures the contribution of electrostatic correlations derived from the mean-spherical approximation of Blum and co-workers,^58,59^ and *F*_ch_ captures the entropic penalty from the formation of chains of charged beads.^55^ This framework applies to arbitrary multicomponent mixtures and has been used to reproduce experimental phase behavior,^57^ making it well suited for identifying the origins of the exclusion of guest polymers in the systems considered here.

Solving the phase–equilibrium conditions^60^ yields global phase diagrams for each guest class (Figure 4A–C). In each diagram, the enclosed colored region denotes the two-phase domain; compositions inside this region split into two phases whose compositions (filled circles) lie on a single tie line (dashed lines). In the absence of guest (ϕ_*g*_ = 0), the system already separates into a coacervate phase (ϕ_*h*_ ≈ 0.25) and a supernatant, consistent with simulations. The grey dashed line represents the process of replacing solvent with guest while keeping the host concentration fixed, mimicking the protocol used in the simulations. Because simulations introduce a small amount of guest (ϕ_*g*_ ≈ 0.01), guest expulsion is predicted whenever this grey path intersects the two-phase region at these low compositions.

**Figure 4:**
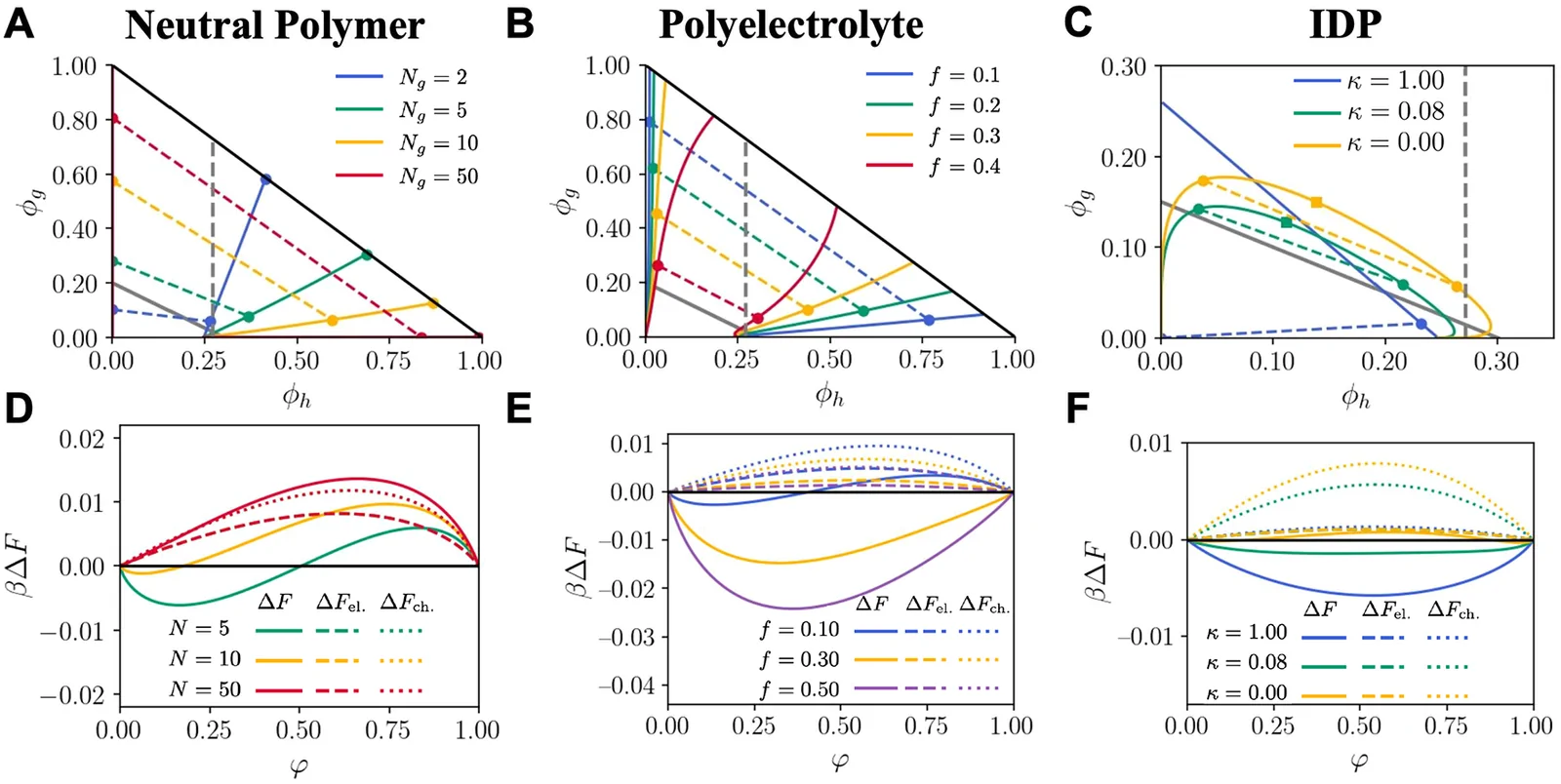
The physical origin of the exclusion of guest polymers and its consequence on the guest encapsulation efficiency. (A–C), Phase diagrams of the host (ϕ_*h*_, *N*_*h*_ = 30) + guest (ϕ_*g*_, *N*_*g*_ = 50, unless indicated otherwise) for each guest considered in this work. Areas enclosed by the solid colored lines represent the two-phase regions where, if the composition of our systems enters this region, a phase split is expected to occur where the composition of the coexisting phases lies on a tie line (examples given by the colored dashed lines). The dashed grey lines represent the process of substituting solvent with guest species, as done in our earlier work. If this line enters the two-phase region, the guest is expelled from the coacervate phase. The solid grey line represents the specific tie line over which the mixing free energies in panels (D–F) are obtained. Not included for simplicity is the three-phase region present in (B), which occurs at low ϕ_*g*_. (D–F), Free energies of mixing, and their respective electrostatic and chain contributions, obtained when traversing the phase diagrams along the solid grey line, where φ denotes the path along the tie line, for each guest considered. Conditions correspond exactly to those used in panels (A–C).

For neutral guests, increasing chain length widens the two-phase region; when *N*_*g*_ = 50, nearly the entire diagram lies within the two-phase domain, as shown in Figure 4A. Along the grey path, the point at which phase separation occurs shifts to progressively smaller guest fractions as *N*_*g*_ increases. This aligns with simulation results: for *N*_*g*_ = 10, ϕ_*g*_ = 0.01 remains in the one-phase region, whereas for *N*_*g*_ = 50 the system expels almost all guests, as indicated by the red tie line linking a guest-free coacervate to a host-free supernatant.

To identify the driving forces, we compute the free-energy change along an arbitrary path (black line in Figure 4A) between a guest-free coacervate (φ = 0) and a coacervate-free mixture (φ = 1). Note that, even if a tangent line can be drawn along this projection, the compositions along this path do not necessarily correspond to the true, globally optimal phases. While we could have chosen a path that connects coexisting phases, doing so in a fair comparison between all systems is unfeasible. Further, this approach allows us to decompose the free energy (Figure 4D) into the electrostatic correlation contribution Δ*F*_el_ and the chain-formation contribution Δ*F*_ch_, where both are strongly unfavorable as *N*_*g*_ increases. Introducing neutral guest dilutes the charge concentration, thereby weakening electrostatic attractions, an energetically costly effect. Moreover, chain formation among like charges is more extended relative to a neutral chain due to electrostatic repulsions. Such conformations are entropically favorable and diluting the charged environment reduces this effect and produces an unfavorable contribution to Δ*F*.

Both mechanisms are insensitive to the guest’s chain length and thus behave analogously to the “enthalpic” term in a Flory–Huggins description, but with the crucial distinction that electrostatic interactions are inherently multi-body and cannot be captured by a single χ-parameter.^61^ These contributions compete with the entropy of mixing, which decreases with increasing chain length of either component. Consequently, once both host or guest chain length exceeds a certain chain length (*N*_*g*_, *N*_*h*_ > 50), the mixing entropy becomes too small to offset the unfavorable electrostatic correlation and chain formation contributions, and neutral guests are universally expelled. Furthermore, this electrostatic exclusion can also be understood using electrostatic blob argument,^45^ where the guest polymers disrupt the electrostatic correlation between neighboring oppositely charged blobs, with a blob size of around 1.7 nm, as detailed in Section S10 of the Supporting Materials.

When the guest carries a small charge fraction *f*, the phase behavior changes qualitatively (Figure 4B). Introducing a weak polyelectrolyte necessarily introduces counterions as well; ϕ_*g*_ therefore represents the combined volume fraction of the guest beads and their neutralizing counterions. As *f* increases, the two-phase region shrinks, and for *f* = 0.50 the grey path no longer intersects the two-phase domain, consistent with simulations, where higher-charge-density guests readily mix with the coacervate.

The free-energy decomposition along the black path in Figure 4B (plotted in Figure 4E) confirms that both Δ*F*_el_ and Δ*F*_ch_ are reduced relative to the neutral case. This is because the guest now introduces some charge, reducing the dilution of the electrostatic interactions. Moreover, the accompanying counterions contribute strongly to the entropy of mixing, further incentivizing mixing. Together, these effects suppress the exclusion of the charged guest observed for neutral guests.

The behavior of intrinsically disordered proteins (IDPs) is more nuanced. The two-phase region is much smaller overall, requiring a zoomed-in axis in Figure 4C. More ordered sequences (κ = 1.0) can even self-coacervate in the absence of host polymer (ϕ_*h*_ = 0). Because these IDPs gain electrostatic cohesion from contacts within their own chains, they mix favorably with the host coacervate, as reflected by the positive-slope tie lines. Only when the sequence becomes sufficiently disordered does the two-phase region begin to expand, and the tie lines invert to indicate expulsion rather than mixing. The more disordered sequences (κ = 0.08 and 0.00) exhibit a re-entrant-like behavior: the two-phase region grows at low guest fractions but closes again at larger ones. The grey path intersects the two-phase domain only when the sequence is fully disordered (κ = 0.00), consistent with the simulation results.

The free energy change contributions in Figure 4F show that, unlike the previous two cases, the electrostatic contribution Δ*F*_el_ is nearly negligible across all IDP sequences. Because the guest introduces fully charged species (even though the net charge is zero), the overall charge density is not diluted, and electrostatic correlations remain largely unchanged. Exclusion of the guest is therefore driven almost entirely by the chain-formation term Δ*F*_ch_.

IDPs differ from standard polyelectrolytes because they form both like-charged and oppositely-charged neighboring pairs. Unlike polyelectrolytes, which only contain like-charged neighbors, IDPs experience favorable local electrostatic attractions that promote locally collapsed conformations, reducing chain entropy. More disordered sequences (smaller κ) contain more such unlike-charge contacts and therefore incur a larger entropic penalty upon forming chains. This penalty is only sufficient to drive phase separation when the sequence is highly disordered, and even then the driving force is weak; a slight increase in charge concentration would increase screening and eliminate the effect, restoring a single-phase system. This explains the re-entrant behavior observed in Figure 4C. Note that the approach used only considers pair-wise interactions. As such, we do not account for any long-range, intramolecular interactions within IDPs.

### Impact on Guest Encapsulation Efficiency

The strong electrostatic exclusion has direct implications for the guest encapsulation efficiency of coacervates, which is crucial for many applications relying on the enrichment of guest molecules. Here, we define guest encapsulation efficiency as the monomer concentration for spontaneous phase separation of guest polymers, beyond which the guest polymers can no longer remain dispersed within the coacervate phase. Thermodynamically, this concentration is the binodal concentration. Since computing the binodal concentration is computationally costly, we instead approximate it using the spinodal concentration, ρ_*m*_^*s*^. Taking the osmotic pressure of guest polymers as the virial expansion of guest density, the spinodal concentration ρ_*m*_^*s*^ relates to the second osmotic virial coefficient *B*_2_ as $\rho_{m}^{s}=-\frac{1}{2B_{2}}$ , where *B*_2_ can be calculated via the guest PMF:^36,62^

2 $$B_{2}=-2\pi \int \left(e^{-\Delta F\left(r\right)/k_{B}T}-1\right)r^{2}dr$$

Figure 5A shows that ρ_*m*_^*s*^ decreases nearly exponentially with increasing guest chain length *N*_*g*_, a phenomenological trend observed within the tested regime 30 < *N*_*g*_ < 150. As such, while smaller molecules *N*_*g*_ < 10 can be easily enriched within the coacervate phase, the encapsulation efficiency drastically decreases for longer chains. For guest polymers with chain length *N*_*g*_ ≥ 50, the spinodal concentration drops to ρ_*m*_^*s*^ ≤ 1.67 × 10^–4^ *M*—a very low encapsulation efficiency compared to the host monomer concentration 5.23*M* in the coacervate. In contrast, in the case of guest polyelectrolyte, its spinodal ρ_*m*_^*s*^ in coacervates increases exponentially with its charge fraction *f* (Figure 5B), suggesting that the encapsulation efficiency can be substantially improved by grafting more charged residues,^63^ consistent with experiments.^26,52,64^

**Figure 5:**
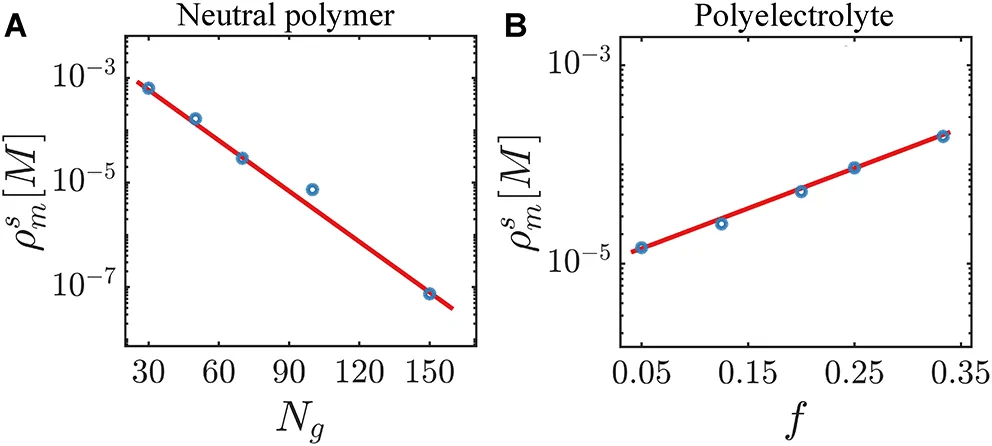
Impact on guest encapsulation efficiency. (A), Spinodal concentration ρ_*m*_^*s*^ of neutral guest polymers in coacervate as a function of their chain length *N*_*g*_ = 30–150. (B) Spinodal concentration ρ_*m*_^*s*^ of guest polyelectrolytes with *N*_*g*_ = 100 in coacervate as a function of their charge density *f* = 5%∼33%. The solid lines represent exponential fits to the data points, indicating an exponential decay of the encapsulation efficiency with chain length for neutral guests and its exponential increase with charge density for guest polyelectrolytes.

## Conclusion

This study demonstrates that the disruption of electrostatic correlations within coacervates can robustly repel guest polymers, even in the absence of chemical incompatibility. The electrostatic exclusion effect elucidated in this work provides a plausible explanation as to why experimentally, coacervates can generally encapsulate small molecules such as short dextran, but strongly exclude large neutral, hydrophilic polymers like long PEG and dextran.^20–22^ Due to the long-range, many-body nature of electrostatic interactions, neutral guest molecules may experience locally favorable interactions with the charged host polymers while still being thermodynamically disfavored overall. Specifically, the incorporation of neutral guests disrupts the long-range electrostatic correlations within the host coacervate, giving rise to an effective unfavorable contribution to the free energy. As the chain length of the neutral guest increases, its translational entropy decreases, eventually leading to the exclusion of sufficiently long chains from the coacervate phase. Because this mechanism originates from many-body electrostatic correlations, it can only be captured through the explicit treatment of electrostatic interactions and lies beyond the scope of conventional Flory–Huggins descriptions. In this work, this exclusion phenomenon manifests as effective guest attraction within coacervates. Meaning, if the supernatant phase exists, the guest species showing effective attraction within the coacervate will be excluded and disperse into the dilute phase. For neutral guest polymers, the exclusion strength increases with guest chain length, leading to exponential decay in encapsulation efficiency. The strong exclusion force can only be diminished by incorporating more than 50% of charges on the guest backbone. As such, while coacervates are known for their efficiency in encapsulating small molecules,^65–68^ encapsulating macromolecules becomes challenging without the addition of charged residues.

For polyampholyte-like intrinsically disordered proteins (IDPs), their effective interactions within coacervates are largely determined by their sequence disorder: IDPs with more disordered sequences experience strict rejection from the coacervate phase, whereas those with blocky charge patterns are dispersed within coacervates. Such sequence-dependent selectivity by the complex coacervate can be explained by the mismatch in guest-host and host–host electrostatic correlations. Scaling theories^38,69^ indicate that the density and charge correlations of blocky polyampholyte self-coacervates closely coincide with those of inter-polyelectrolyte complex coacervates. Recent RPA studies further clarify that, over a specific range of sequence blockiness, the structural correlations in self- and complex coacervates follow the same limiting behavior.^70,71^ This similarity in correlation properties between the blocky polyampholyte self-coacervates and complex coacervates would minimize the energetic and entropic cost of guest encapsulation, thereby promoting mixing. In contrast, guest IDPs with disordered sequences would be excluded from the dense coacervate phase as a result of the disruption of the intrinsic electrostatic correlations in the coacervate matrix.

At first glance, one might expect the driving force behind this phenomena to be purely energy-driven, since expelling the guest maximizes the strength of the electrostatic correlations that stabilize the host coacervate. However, our theoretical analysis reveals a more nuanced thermodynamic picture. Expulsion of guest polymers is driven by the combined effect of two contributions. First, an energetic penalty is associated with weakening electrostatic correlations when the guest dilutes the local charge density. Second, an entropic penalty is associated with the loss of chain configurational freedom when charged beads are assembled into chains. Both contributions oppose mixing and compete directly against the entropy of mixing. Whether exclusion occurs, therefore, depends on the balance between these effects. Neutral polymers, for example, strongly weaken electrostatic correlations while also reducing the favorable chain entropy of the charged host, through the weakened electrostatic interactions. This leads to a strong driving force for excluding the guest from the coacervates. Guest polyelectrolytes, in contrast, introduce additional charged species and counterions, mitigating both penalties and restoring miscibility at higher charge fractions. This mechanism may explain why “supercharged” guest proteins can be encapsulated by complex coacervates, despite severely breaking the overall macromolecular charge neutrality of the host coacervate phase.^23–25^ Our results also suggest that, for guest IDPs, beyond similarities in charge sequence with the host polyelectrolytes, the driving force for partitioning can arise from differences in conformational entropy.

As the focus of this study is to investigate the physical principle of how the densely charged coacervate environment regulates guest behavior, our simulations and theories incorporate some necessary assumptions. For example, to highlight the role of electrostatics, we set χ = 0 across all species, representing a good solvent for both the host and guest species. Therefore, our simulations do not capture the chemical specificity and hydrogen-bonding networks in real systems. In addition, our liquid-state theory is based on a mean-field approximation and does not account for detailed multi-body interactions. Furthermore, our models use relatively short flexible chains, which may miss possible long-range intramolecular IDP correlations due to specific many-body effects. Bridging the gaps between these simplifications and the complexity of real systems, especially how the specific short-range interactions couple with long-range electrostatics to influence guest encapsulation in real systems, presents a compelling challenge for future study.

Our work elucidates a fundamental mechanism whereby coacervates regulate guest macromolecules, with significant implications for rational design of coacervate systems essential for synthetic and biological applications. For example, understanding how the nanoscale charge correlation of the host coacervate matrix regulates the phase behavior of guest polymers provides valuable insights for spatial organization of multiphase biocondensates, rational design of smart nanoreactors, and targeted drug delivery vehicles.

## Methods

### Simulation Methodology

In this study, we employ the Gaussian Core Model with Smeared Electrostatics (GCMe)^72^ to perform coarse-grained simulations to investigate coacervate systems. The monomers of both host and guest chains, counterions, and solvent molecules (water) are represented as coarse-grained beads, with each bead having a Gaussian distribution in mass and charge density around its center. We set the excluded volume interactions to be the same among all species, representing a good solvent for all the polymers. The Bjerrum length *l*_B_ is set to match the dielectric environment of water at room temperature. Other simulation details, including the interactions parameter, the preparation of the complex coacervate, and the PMF calculation, are included in the Supporting Materials.^73^

### Experimental Methods

In the experiments, complex coacervates are prepared by mixing oppositely charged polymers at a 1:1 charge ratio in HEPES buffer (to ensure a final pH of 7.0 ± 0.2). The mixing behavior of two different complex coacervates is visualized using microscopy with fluorescently labeled components. The binding constant between two coacervate species is obtained from the ITC measurement. Other experimental details, including the choice of materials, sample preparation, measure procedure, are included in the Supporting Materials.^73^

### Theoretical Analysis

The theoretical calculations are carried out using liquid-state theory, where the free energy explicitly include the effects of excluded volume, chain connectivity and electrostatic correlations. The phase diagrams for each guest category are obtained from solving the phase-equilibrium conditions.

## Supplementary Material



## Data Availability

All data is included in the article and/or in the Supporting Information.^73^ Source data were available from the corresponding author upon request. The codes used in this study are available from the corresponding author on request.

## Notes

Simulation details, preparation of the complex coacervate matrices, and the PMF calculation between two guest polymers; guest behavior within coacervates with *N*_*h*_ = 100 and within the dilute phase; aggregation of 50% charged IDP guests; PMF of guests in different solvents and the decomposition of the PMF in coacervate; compression of a single chain within the coacervate phase; experimental materials, fluorescent labeling, confocal microscopy, and isothermal titration calorimetry; and the electrostatic blob interpretation of the electrostatic exclusion, including Figures S1–S9 (PDF)

## References

[1] Banani S. F., Lee H. O., Hyman A. A., Rosen M. K. (2017). Biomolecular condensates:
organizers of cellular biochemistry. Nat. Rev.
Mol. Cell Biol..

[2] Deshpande S., Brandenburg F., Lau A., Last M. G., Spoelstra W. K., Reese L., Wunnava S., Dogterom M., Dekker C. (2019). Spatiotemporal
control of coacervate formation within liposomes. Nat. Commun..

[3] Abbas M., Lipiński W. P., Wang J., Spruijt E. (2021). Peptide-based coacervates
as biomimetic protocells. Chem. Soc. Rev..

[4] Antifeeva I. A., Fonin A. V., Fefilova A. S., Stepanenko O. V., Povarova O. I., Silonov S. A., Kuznetsova I. M., Uversky V. N., Turoverov K. K. (2022). Liquid-liquid phase separation as
an organizing principle of intracellular space: Overview of the evolution
of the cell compartmentalization concept. Cell.
Mol. Life Sci..

[5] Johnston A. R., Perry S. L., Ayzner A. L. (2021). Associative
phase separation of aqueous *π*-conjugated polyelectrolytes
couples photophysical
and mechanical properties. Chem. Mater..

[6] Xu C., Martin N., Li M., Mann S. (2022). Living material assembly
of bacteriogenic protocells. Nature.

[7] Cook A. B., Novosedlik S., van Hest J. C. (2023). Complex coacervate materials as artificial
cells. Acc. Mater. Res..

[8] Black K. A., Priftis D., Perry S. L., Yip J., Byun W. Y., Tirrell M. (2014). Protein encapsulation via polypeptide
complex coacervation. ACS Macro Lett..

[9] Blocher
McTigue W. C., Perry S. L. (2020). Protein encapsulation using complex
coacervates: what nature has to teach us. Small.

[10] Schoonen L., Van Hest J. C. (2016). Compartmentalization approaches in
soft matter science:
from nanoreactor development to organelle mimics. Adv. Mater..

[11] Cao S., Ivanov T., de Souza Melchiors M., Landfester K., Caire da Silva L. (2023). Controlled
membrane transport in polymeric biomimetic
nanoreactors. ChemBioChem.

[12] Sing C. E., Perry S. L. (2020). Recent progress in the science of complex coacervation. Soft Matter.

[13] Rumyantsev A. M., Jackson N. E., De Pablo J. J. (2021). Polyelectrolyte complex coacervates:
Recent developments and new frontiers. Annu.
Rev. Condens. Matter Phys..

[14] Tang T.-Y. D., Antognozzi M., Vicary J. A., Perriman A. W., Mann S. (2013). Small-molecule
uptake in membrane-free peptide/nucleotide protocells. Soft Matter.

[15] Tang T.-Y. D., Hak C. R. C., Thompson A. J., Kuimova M. K., Williams D., Perriman A. W., Mann S. (2014). Fatty acid membrane assembly on coacervate
microdroplets as a step towards a hybrid protocell model. Nat. Chem..

[16] Love C., Steinkühler J., Gonzales D. T., Yandrapalli N., Robinson T., Dimova R., Tang T.-Y. D. (2020). Reversible pH-responsive
coacervate formation in lipid vesicles activates dormant enzymatic
reactions. Angew. Chem..

[17] Roberts S., Dzuricky M., Chilkoti A. (2015). Elastin-like
polypeptides as models
of intrinsically disordered proteins. FEBS Lett..

[18] Nakashima K. K., Baaij J. F., Spruijt E. (2018). Reversible
generation of coacervate
droplets in an enzymatic network. Soft Matter.

[19] Martin N., Li M., Mann S. (2016). Selective
uptake and refolding of globular proteins
in coacervate microdroplets. Langmuir.

[20] Marianelli A. M., Miller B. M., Keating C. D. (2018). Impact
of macromolecular crowding
on RNA/spermine complex coacervation and oligonucleotide compartmentalization. Soft Matter.

[21] Biswas S., Hecht A. L., Noble S. A., Huang Q., Gillilan R. E., Xu A. Y. (2023). Understanding the impacts of molecular and macromolecular crowding
agents on protein-polymer complex coacervates. Biomacromolecules.

[22] Moreau N. G., Martin N., Gobbo P., Tang T.-Y. D., Mann S. (2020). Spontaneous
membrane-less multi-compartmentalization via aqueous two-phase separation
in complex coacervate micro-droplets. Chem.
Commun..

[23] Obermeyer A. C., Mills C. E., Dong X.-H., Flores R. J., Olsen B. D. (2016). Complex
coacervation of supercharged proteins with polyelectrolytes. Soft Matter.

[24] Cummings C. S., Obermeyer A. C. (2018). Phase separation behavior of supercharged proteins
and polyelectrolytes. Biochemistry.

[25] Ahn S. Y., Obermeyer A. C. (2024). Selectivity
of Complex Coacervation in Multiprotein
Mixtures. JACS Au.

[26] McTigue W. C. B., Perry S. L. (2019). Design rules for
encapsulating proteins into complex
coacervates. Soft Matter.

[27] Overbeek J. T. G., Voorn M. J. (1957). Phase separation
in polyelectrolyte solutions. Theory
of complex coacervation. J. Cell. Comp. Physiol..

[28] Kudlay A., de la Cruz M. O. (2004). Precipitation
of oppositely charged polyelectrolytes
in salt solutions. J. Chem. Phys..

[29] Kudlay A., Ermoshkin A. V., de La Cruz M. O. (2004). Complexation of oppositely charged
polyelectrolytes: effect of ion pair formation. Macromolecules.

[30] Qin J., Priftis D., Farina R., Perry S. L., Leon L., Whitmer J., Hoffmann K., Tirrell M., De Pablo J. J. (2014). Interfacial
tension of polyelectrolyte complex coacervate phases. ACS Macro Lett..

[31] Qin J., de Pablo J. J. (2016). Criticality and connectivity in macromolecular charge
complexation. Macromolecules.

[32] Rumyantsev A. M., Jackson N. E., Yu B., Ting J. M., Chen W., Tirrell M. V., De Pablo J. J. (2019). Controlling
complex coacervation
via random polyelectrolyte sequences. ACS Macro
Lett..

[33] Zhang P., Shen K., Alsaifi N. M., Wang Z.-G. (2018). Salt Partitioning
in Complex Coacervation of Symmetric Polyelectrolytes. Macromolecules.

[34] Zhang P., Alsaifi N. M., Wu J., Wang Z.-G. (2018). Polyelectrolyte
complex coacervation: Effects of concentration asymmetry. J. Chem. Phys..

[35] Shen K., Wang Z.-G. (2018). Polyelectrolyte
chain structure and solution phase
behavior. Macromolecules.

[36] Chen S., Wang Z.-G. (2022). Driving force and
pathway in polyelectrolyte complex
coacervation. Proc. Natl. Acad. Sci. U.S.A..

[37] Lee J., Popov Y. O., Fredrickson G. H. (2008). Complex coacervation: A field theoretic
simulation study of polyelectrolyte complexation. J. Chem. Phys..

[38] Delaney K. T., Fredrickson G. H. (2017). Theory
of polyelectrolyte complexation-Complex coacervates
are self-coacervates. J. Chem. Phys..

[39] Friedowitz S., Qin J. (2021). Reversible ion binding for polyelectrolytes with adaptive conformations. AIChE J..

[40] Jha P. K., Desai P. S., Li J., Larson R. G. (2014). pH and salt effects
on the associative phase separation of oppositely charged polyelectrolytes. Polymers.

[41] Ghasemi M., Larson R. G. (2021). Role of electrostatic interactions in charge regulation
of weakly dissociating polyacids. Prog. Polym.
Sci..

[42] Borue V. Y., Erukhimovich I. Y. (1990). A statistical
theory of globular polyelectrolyte complexes. Macromolecules.

[43] Lytle T. K., Sing C. E. (2017). Transfer matrix theory of polymer complex coacervation. Soft Matter.

[44] Knoerdel A. R., Blocher McTigue W. C., Sing C. E. (2021). Transfer matrix model of pH effects
in polymeric complex coacervation. J. Phys.
Chem. B.

[45] Fang Y. N., Rumyantsev A. M., Neitzel A. E., Liang H., Heller W. T., Nealey P. F., Tirrell M. V., de Pablo J. J. (2023). Scattering evidence
of positional charge correlations in polyelectrolyte complexes. Proc. Natl. Acad. Sci. U.S.A..

[46] Chen X., Chen E.-Q., Shi A.-C., Yang S. (2021). Multiphase coacervates
driven by electrostatic correlations. ACS Macro
Lett..

[47] Chen X., Chen E.-Q., Yang S. (2023). Multiphase coacervation of polyelectrolytes
driven by asymmetry of charged sequence. Macromolecules.

[48] van
der Schoot P. (1998). Protein-induced collapse of polymer chains. Macromolecules.

[49] Asakura S., Oosawa F. (1954). On interaction between two bodies immersed in a solution
of macromolecules. J. Chem. Phys..

[50] Asakura S., Oosawa F. (1958). Interaction between
particles suspended in solutions
of macromolecules. J. Polym. Sci..

[51] Das R. K., Pappu R. V. (2013). Conformations of
intrinsically disordered proteins
are influenced by linear sequence distributions of oppositely charged
residues. Proc. Natl. Acad. Sci. U.S.A..

[52] Agrawal A., Fang Y. N., Rizvi S., Noor Azman N. F., Tirrell M. V., Karim A., Neitzel A. E. (2025). Charge
Density Mismatch
Drives Demixing in Multicomponent Polyelectrolyte Complexes. Macromolecules.

[53] Nott T. J., Petsalaki E., Farber P., Jervis D., Fussner E., Plochowietz A., Craggs T. D., Bazett-Jones D. P., Pawson T., Forman-Kay J. D., Baldwin A. (2015). Phase transition of
a disordered nuage protein generates environmentally responsive membraneless
organelles. Mol. Cell.

[54] Madinya J. J., Chang L.-W., Perry S. L., Sing C. E. (2020). Sequence-dependent
self-coacervation in high charge-density polyampholytes. Mol. Syst. Des. Eng..

[55] Jiang J. W., Blum L., Bernard O., Prausnitz J. M. (2001). Thermodynamic
properties and phase equilibria of charged hard sphere chain model
for polyelectrolyte solutions. Mol. Phys..

[56] Zhang P., Alsaifi N. M., Wu J., Wang Z.-G. (2016). Salting-Out and
Salting-In of Polyelectrolyte Solutions: A Liquid-State Theory Study. Macromolecules.

[57] Ylitalo A. S., Balzer C., Zhang P., Wang Z.-G. (2021). Electrostatic
Correlations
and Temperature-Dependent Dielectric Constant Can Model LCST in Polyelectrolyte
Complex Coacervation. Macromolecules.

[58] Blum L. (1975). Mean spherical
model for asymmetric electrolytes. Mol. Phys..

[59] Blum L., Hoeye J. S. (1977). Mean spherical model
for asymmetric electrolytes. 2.
Thermodynamic properties and the pair correlation function. J. Phys. Chem. A.

[60] Walker P. J., Yew H.-W., Riedemann A. (2022). Clapeyron.jl: An Extensible, Open-Source
Fluid Thermodynamics Toolkit. Ind. Eng. Chem.
Res..

[61] Walker P. J., Wang Z.-G. (2025). Anomalous Phase Behavior of Polymer–Ionic Liquid
Mixtures. J. Phys. Chem. B.

[62] Wang Z.-G. (2017). 50th anniversary
perspective: Polymer conformation - A pedagogical review. Macromolecules.

[63] Peng B., Muthukumar M. (2015). Modeling competitive substitution
in a polyelectrolyte
complex. J. Chem. Phys..

[64] Blocher W. C., Perry S. L. (2017). Complex coacervate-based
materials for biomedicine. WIREs Nanomed. Nanobiotechnol..

[65] Johnson N. R., Wang Y. (2014). Coacervate delivery systems for proteins and small molecule drugs. Expert Opin. Drug Delivery.

[66] Lv K., Perriman A. W., Mann S. (2015). Photocatalytic
multiphase micro-droplet
reactors based on complex coacervation. Chem.
Commun..

[67] Anraku Y., Kishimura A., Kamiya M., Tanaka S., Nomoto T., Toh K., Matsumoto Y., Fukushima S., Sueyoshi D., Kano M. R. (2016). Systemically injectable enzyme-loaded polyion complex vesicles as
in vivo nanoreactors functioning in tumors. Angew. Chem., Int. Ed..

[68] Egbeyemi O. I., Hatem W. A., Kober U. A., Lapitsky Y. (2024). Transforming the stability,
encapsulation, and sustained release properties of calcium alginate
beads through gel-confined coacervation. Langmuir.

[69] Shusharina N. P., Zhulina E. B., Dobrynin A. V., Rubinstein M. (2005). Scaling theory
of diblock polyampholyte solutions. Macromolecules.

[70] Rumyantsev A. M., Jackson N. E., Johner A., De Pablo J. J. (2021). Scaling theory of
neutral sequence-specific polyampholytes. Macromolecules.

[71] Rumyantsev A. M., Johner A. (2023). Salt-added solutions
of Markov polyampholytes: Diagram
of states, antipolyelectrolyte effect, and self-coacervate dynamics. Macromolecules.

[72] Ye B. B., Chen S., Wang Z.-G. (2024). GCMe: Efficient
implementation of
the Gaussian core model with smeared electrostatic interactions for
molecular dynamics simulations of soft matter systems. J. Chem. Theory Comput..

[73] See Supplemental Material at https://pubs.acs.org/doi/10.1021/acsnano.6c06413 for additional details.

