# Supplementary material for: Origin of the Anomalous Guest Polymer Selectivity in Complex Coacervates
Source: ACS Nano. 2026 Sep 29;20(40):27054–65. doi: 10.1021/acsnano.6c06413 (PMC13671173; doi:10.1021/acsnano.6c06413)
Supplement: Supplementary file 1 [file nn6c06413_si_001.pdf]

Supplementary Information for Origin of the Anomalous Guest Polymer Selectivity in Complex Coacervates

Zongpei Wu[*,1], Júlia Bonesso Sabadini[*,2,4], Pierre J. Walker[*,†,3], Watson Loh[2], Zhen-Gang Wang[3], Sarah L. Perry[†,4], and Shensheng Chen[†,1]

[1]Department of Chemical and Biological Engineering, The Hong Kong University of Science and Technology, Clear Water Bay, Hong Kong, China
[2]Institute of Chemistry, University of Campinas (UNICAMP), P.O. Box 6154, 13083-970, Campinas, São Paulo, Brazil
[3]Division of Chemistry and Chemical Engineering, California Institute of Technology, Pasadena, California 91125, USA
[4]Department of Chemical and Biomolecular Engineering, University of Massachusetts Amherst, Amherst, Massachusetts 01003, United States

∗These authors contributed equally to this work
†Email: pjwalker@caltech.edu; perrys@engin.umass.edu; shensheng@ust.hk

This PDF file includes:

Supplementary sections S1 to S10
Figs. S1 to S9
References

S1 Simulation Details

In this study, we employ the Gaussian Core Model with Smeared Electrostatics (GCMe) [1] to perform coarse-grained simulations to investigate coacervate systems. Following the default setup of the GCMe model, we set the reduced number density to $\rho = 2.5/\sigma_0^3$, where $\sigma_0 \approx 0.64$nm is the size of coarse-grained beads for all the species (host polyelectrolyte monomers, guest chain monomers, counterions, and solvent molecules) [1]. The excluded volume interaction between bead i and j separated by distance r_{ij} is modeled by a Gaussian soft-core repulsive potential:

$$U_{\mathrm{ex}}(r_{ij}) = A_{ij}\left(\frac{3}{2\pi\sigma_{ij}^2}\right)^{3/2} \exp\left(-\frac{3r_{ij}^2}{2\sigma_{ij}^2}\right) \tag{S1}$$

Here, we set $A_{ij} = 25.2k_BT\sigma_0^3$ the same for all beads, where k_BT is the thermal energy at room temperature, $\sigma_i = \sigma_j = 0.5\sigma_0$ is the mass smearing radii of coarse-grained beads, and $\sigma_{ij} = \sqrt{\sigma_i^2 + \sigma_j^2} = 0.71\sigma_0$. Since A_{ij} is the same for all the beads, there are no differences in hydrophobicity across species.

Long-range electrostatic interaction between two charged beads follows a modified, Gaussian-core Coulomb potential:

$$U_{\mathrm{elec}}(r_{ij}) = \frac{e^2}{4\pi\epsilon_0\epsilon_r r_{ij}} \mathrm{erf}\left(\frac{\pi^{1/2} r_{ij}}{2^{1/2} a_{ij}}\right), \tag{S2}$$

where $a_{ij} = \sqrt{a_i^2 + a_j^2} = 0.71\sigma_0$, $a_i = a_j = 0.5\sigma_0$ is the charge smearing radii, ϵ_0 is the vacuum permittivity, and ϵ_r is the relative permittivity of water at room temperature. The corresponding Bjerrum length $l_B = \frac{e^2}{4\pi\epsilon_0\epsilon k_BT} \approx 1.1\sigma_0$ is set to match the dielectric environment of water at room temperature.

Neighboring polymer monomers along a single chain (including polyion and guest polymer chain) are connected via a harmonic bond potential:

$$E_{\mathrm{bond}} = \frac{1}{2}K_{\mathrm{bond}}(r-b)^2, \tag{S3}$$

where $K_{\mathrm{bond}} = 100k_BT/\sigma_0^2$ is the bond strength. For fully charged polyelectrolytes, the electrostatic strength is $l_B/b \approx 1.0$, falling into intermediately charge regime.

All simulations are performed in a simulation box with a dimension $50\sigma_0 \times 50\sigma_0 \times 50\sigma_0$, using the LAMMPS [2] platform. The characteristic time scale is given by $\tau_0 = \sqrt{m_0\sigma_0^2/(k_BT)} = 7.8$ps, where $m_0 \approx 72$g/mol to represent to coarse-grained level of one water bead contains 4 water molecules. The integration time step is set to $\delta t = 0.01\tau_0$.

S2 Prepare Complex Coacervates in Simulation

We obtain the coacervate compositions from liquid-liquid phase separation (LLPS) of polycation and polyanion mixture in a $120\sigma_0 \times 30\sigma_0 \times 30\sigma_0$ simulation box (Fig. S1). In this study, we investigate a coacervate matrix with host chain lengths of $N_h = 30$ and $N_h = 100$. For the coacervate with host chain length $N_h = 30$, the LLPS system contains $n_c = n_a = 500$ fully charged polycations and polyanions, with 2700 small ions and the remaining water beads initially disperse within the simulation box. For the coacervate system with $N_h = 100$, we set $n_c = n_a = 200$, together with the same number of small ions as before. Both systems are equilibrated for $3 \times 10^4\tau_0$ with fixed center-of-mass of the polyions at $x = 60\sigma_0$ (the snapshots of Fig. S1 A and B).

We quantify the equilibrium composition by computing the density profile along the x direction. Specifically, we divide the simulation box into 120 bins of thickness $1\sigma_0$ along the x direction and count the polyions, water, and small ions, respectively in the bins. For $N_h = 30$, the coacervate contains around 33% polyions, 66% water, and 1% small ions, while coacervate with $N_h = 100$ contains 38% polyions, 61% water, and 1% small ions (Fig. S1 A and B).

Based on the equilibrium profiles from these LLPS simulations, we build bulk coacervate systems in simulation box with dimensions $50\sigma_0 \times 50\sigma_0 \times 50\sigma_0$ by replicating the coacervate compositions for $N_h = 30$

and $N_h = 100$. An example of the resulting coacervate matrix for $N_h = 30$ is shown in Fig. 1A in the main text.

Guest species— including neutral polymers, locally charged polyelectrolytes, and intrinsically disordered proteins (IDPs) — are initially evenly distributed within the coacervate matrices. In each simulation, we study the guest behavior by putting $0.3\% \sim 1.6\%$ guest polymers within the coacervate matrix, with the assumption that they do not significantly alter the properties of the bulk coacervates. The guest polymers are inserted by replacing the solvent particles while maintaining the overall number density of the system. The charged density of the guest polyelectrolytes f is defined as the fraction of the charged monomers, which are evenly distributed along the chain. For example, $f = 33\%$ corresponds to one charged monomer per three neighboring monomers along the chain.

Fully-charged IDPs are prepared by carefully positioning equal number of positive and negative charges along a backbone of 50 monomers. In this work, we use the order parameter κ introduced by Das and Pappu [3] to characterize the IDP charge sequences . Following the standard procedure, we calculate κ by dividing the chain into N_s overlapping segments of size s. For each segment, we first calculate its charge asymmetry as $\lambda_i = \frac{(f_+ - f_-)_i^2}{(f_+ + f_-)_i}$, where f_+ and f_- is the fraction of positively and negatively charged monomers within segment i. We then quantify the squared deviation from perfect charge segregation as $\delta = \frac{\sum_i^{N_\mathrm{s}} (\lambda_i - \lambda)^2}{N_\mathrm{s}}$, where λ is the overall charge asymmetry defined as $\lambda = \frac{(f_+ - f_-)^2}{(f_+ + f_-)}$. Here, we use the deviation from δ_max (δ_max corresponding to the diblock sequence where one half of the chain is positively charged and the other half is negatively charged) to define $\kappa = 1 - \delta/\delta_\mathrm{max}$, ensuring $0 \le \kappa \le 1$. We calculate κ using two segment sizes: $s = 5$ and $s = 6$ and take the average of these two values as the final κ for each charged blockiness sequence variant [3]. In Fig. 1D of the main text, the $\kappa = 1.0$ means fully segregated charges (all positive followed by all negative), where $\kappa = 0.0009$ corresponds to alternative positive-negative distribution.

For any charged guest, additional counterions matching the guest charges are introduced to maintain charge neutrality. For each system, we run $2 \times 10^5 \tau_0$ to reach equilibrium state.

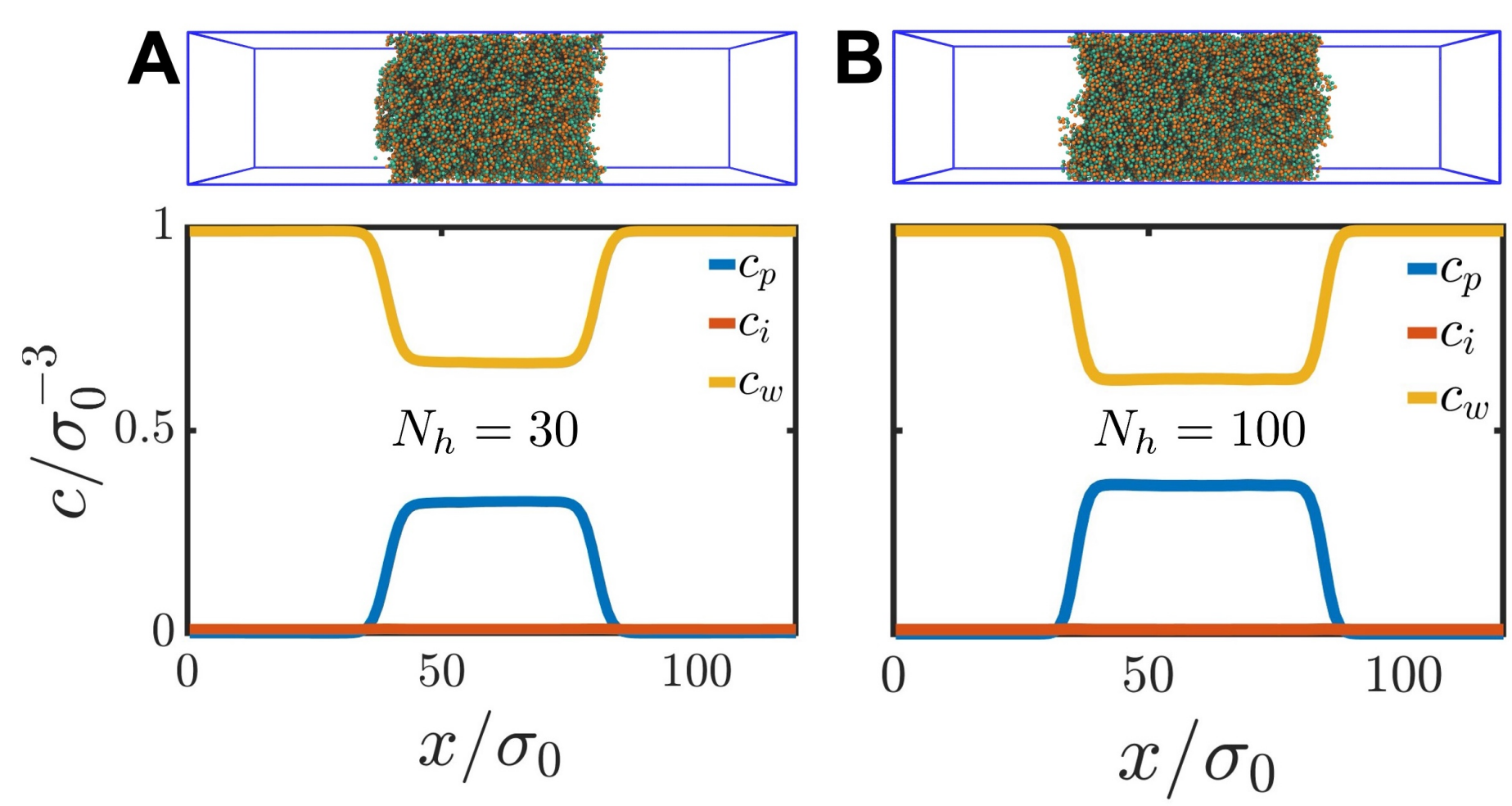

Figure S1: The composition of the coacervate matrix obtained from liquid-liquid phase separation between oppositely charged polyelectrolytes with symmetric chain length $N_h = 30$ (**A**) and $N_h = 100$ (**B**), respectively. c_p, c_i, c_w represent respectively the fractions of polymer, small ions, and water. The upper panels are the snapshots, where the green and orange beads represent the polyanions and polycations, respectively. The water molecules and counterions are omitted for clarity.

S3 PMF Calculation Between Two Guest Polymers

In this study, the interaction between two guest molecules is quantified by their potential of mean force (PMF) ΔF as a function of their center-of-mass distance Δr. We calculate the PMF profiles by using the

adaptive bias force (ABF) algorithm [2, 4] implemented in the LAMMPS platform. The range of Δr is $0\sigma_0 \sim 15\sigma_0$. To improve the sampling efficiency and accuracy, this distance range is partitioned into five continuous sampling windows: $0\sigma_0 \sim 1\sigma_0$, $1\sigma_0 \sim 3\sigma_0$, $3\sigma_0 \sim 6\sigma_0$, $6\sigma_0 \sim 10\sigma_0$, $10\sigma_0 \sim 15\sigma_0$ [5]. Each window is further divided into smaller bins with equal width of $0.1\sigma_0$. The PMF in each window has run for $1 \times 10^6 \tau_0$ to ensure convergence. All the PMF calculations are performed within a $24\sigma_0 \times 12\sigma_0 \times 12\sigma_0$ simulation box, with two guest chains (and their counterions, if any) in the respective environments (coacervate phase or dilute phase).

S4 Guest Behavior Within Coacervate with $N_h = 100$

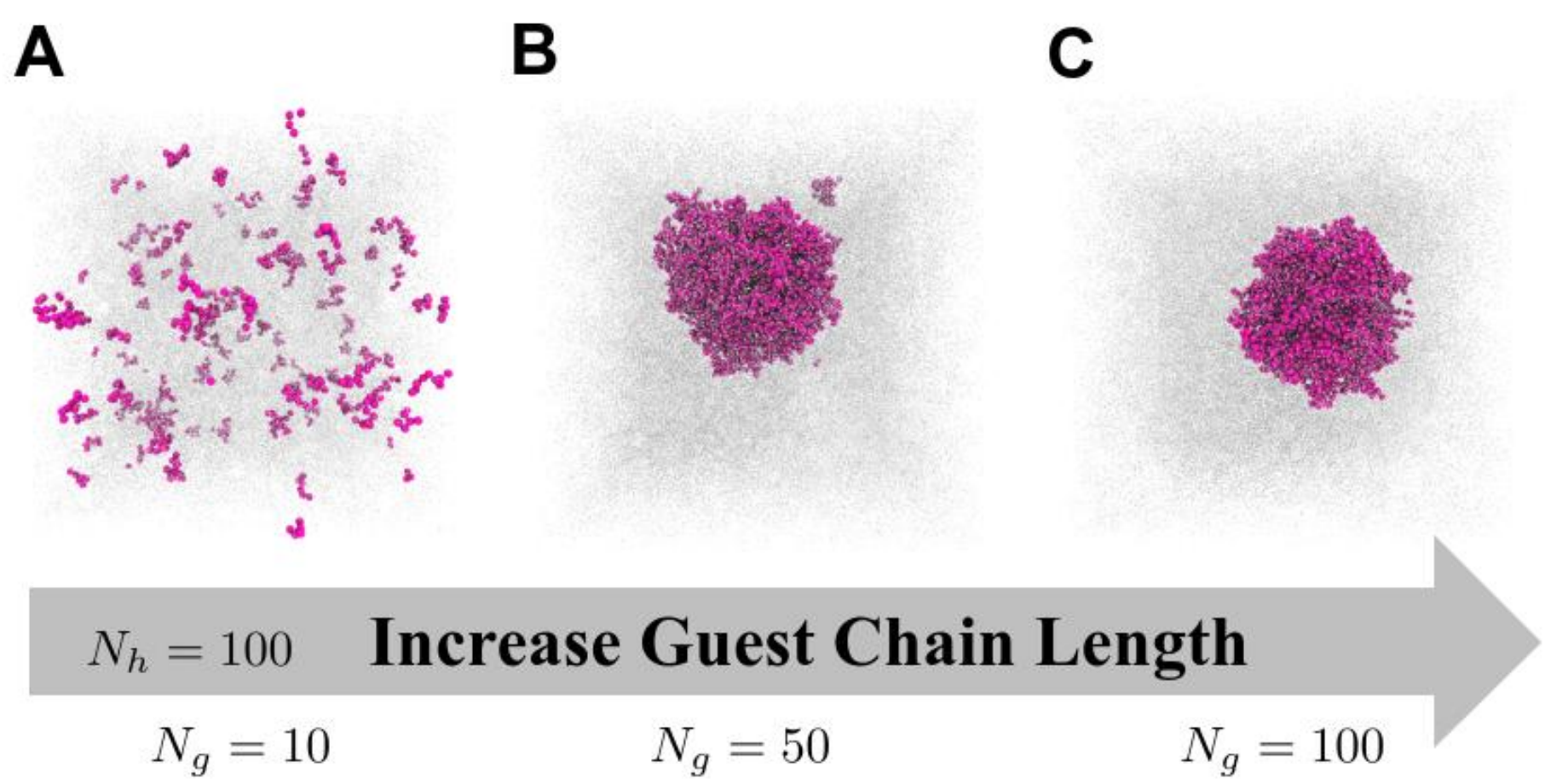

Figure S2: The behavior of neutral guest polymers with different chain length (**A**, $N_g = 10$; **B**, $N_g = 50$; **C**, $N_g = 100$) within the coacervate matrix with $N_h = 100$. Gray and magenta beads represent monomers of the background coacervate matrix and neutral monomers, respectively. The water and counterions are omitted for clarity.

Figure S2 shows the neutral polymer behavior in the coacervate matrix of $N_h = 100$. Similar to the case of $N_h = 30$ detailed in the main text, the guest polymers exhibit apparent aggregation behavior for chain length $N_g \geq 50$. Notably, the neutral polymer can spontaneously aggregate even for $N_g < N_h$. Therefore, the origin of this guest aggregation is not from the classical depletion effect, which requires $N_g \gg N_h$.

S5 Guest Behavior Within Dilute Phase

To highlight the electrostatic exclusion effect is a unique phenomenon in the coacervate phase, we simulate the guest behavior in the dilute phase environment as a comparison. In the dilute phase, both neutral polymers and polyelectrolytes exhibit dispersed distribution due to repulsive intermolecular interactions (Fig, 2G in main texts), as shown in Fig. S3A, B. This is opposite compared to the cases in the coacervate phase, where the neutral polymers and low-charge-density polyelectrolytes show universal aggregation behavior (Fig. 1B, C in the main text). For guest IDPs within the dilute phase, high κ sequence such as $\kappa = 1.0$ first undergo self-coacervation to form a small globule, then aggregate to a larger compact globule (Fig. S3D). However, they become repulsive in the coacervate phase, as detailed in Fig. 2 in the main text. Low κ such as $\kappa = 0.0009$ are repulsive and remain dispersed within the dilute phase (Fig. S3C), while they aggregate within the coacervate phase. Clearly, all the guest chains, including neutral polymers, polyelectrolytes, and IDPs, behave drastically different in the coacervate phase compare to that in the dilute phase.

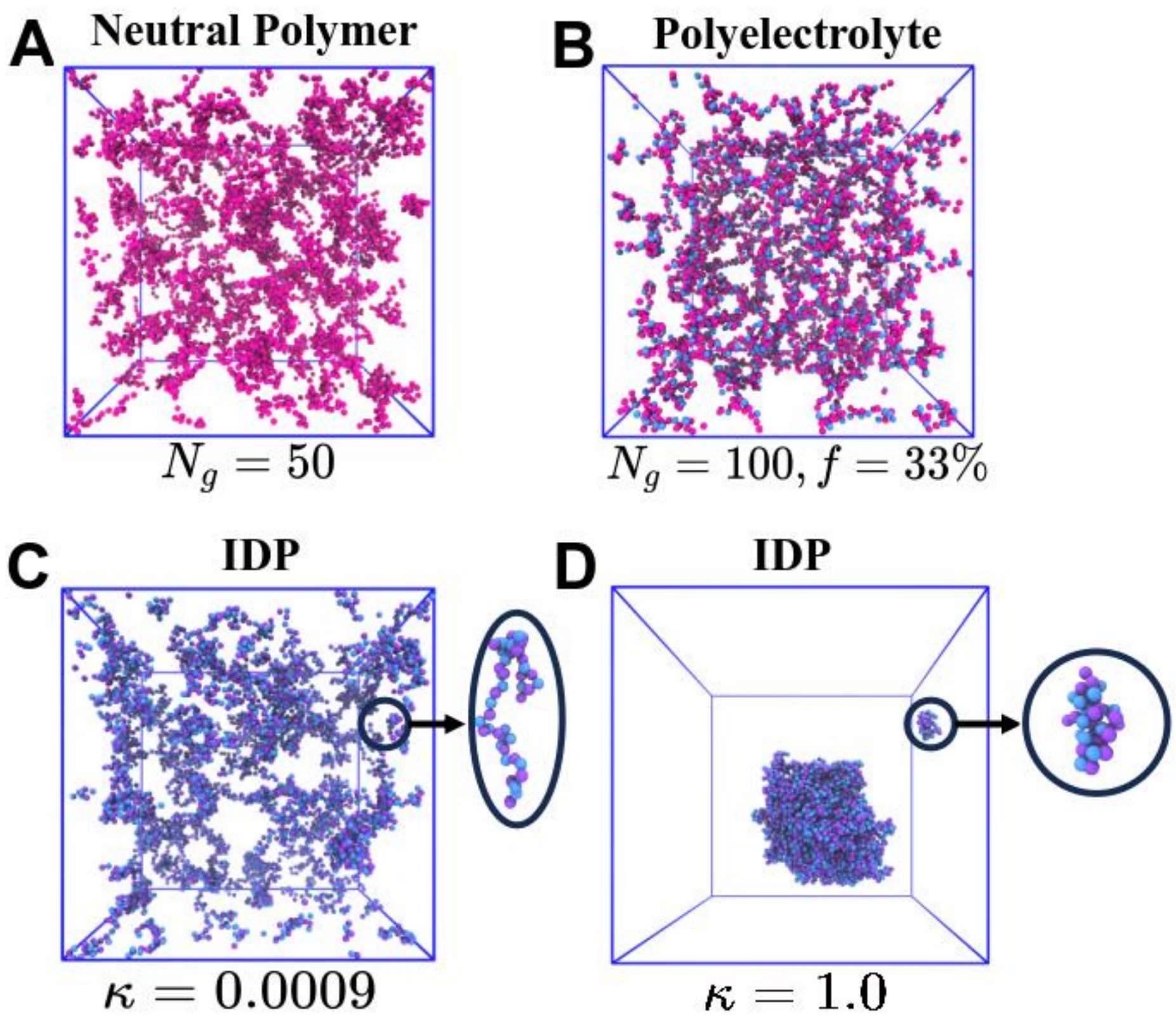

Figure S3: The behavior of around 1% guest polymers in the dilute phase. **A**, Neutral polymer with $N_g = 50$; **B**, Polyelectrolyte with $N_g = 100$ and $f = 33\%$; **C** and **D**, IDP with $\kappa = 0.0009$ and $\kappa = 1.0$, the insets show representative equilibrium configurations of single-chain conformations. Magenta, blue, and purple represent the neutral, negatively charged, and positively charged monomers, respectively. The water is omitted for clarity.

S6 The Behavior for Aggregation of 50% Charged IDP Guest

While fully charged IDPs with very low κ (about 0.014) remain dispersed within the coacervate matrix, biologically relevant IDPs often exhibit aggregation and compartmentalization. We suppose this is because, in real IDP systems, not every monomer is charged, and we expected more prominent IDP aggregations within the coacervate. To this end, we examine IDPs chain length of $N_g = 100$, where half of the monomers carry equal amount (25) of positive and negative charges.

Figure. S4B shows that for IDPs with charged density $f = 50\%$, strong aggregation appears for sequence with $\kappa = 0.14$, while the same κ in the fully-charged IDPs will exhibit dispersed distribution. Thus, aggregations will be more common for real IDPs whose charged density is generally lower than the ones in the main text.

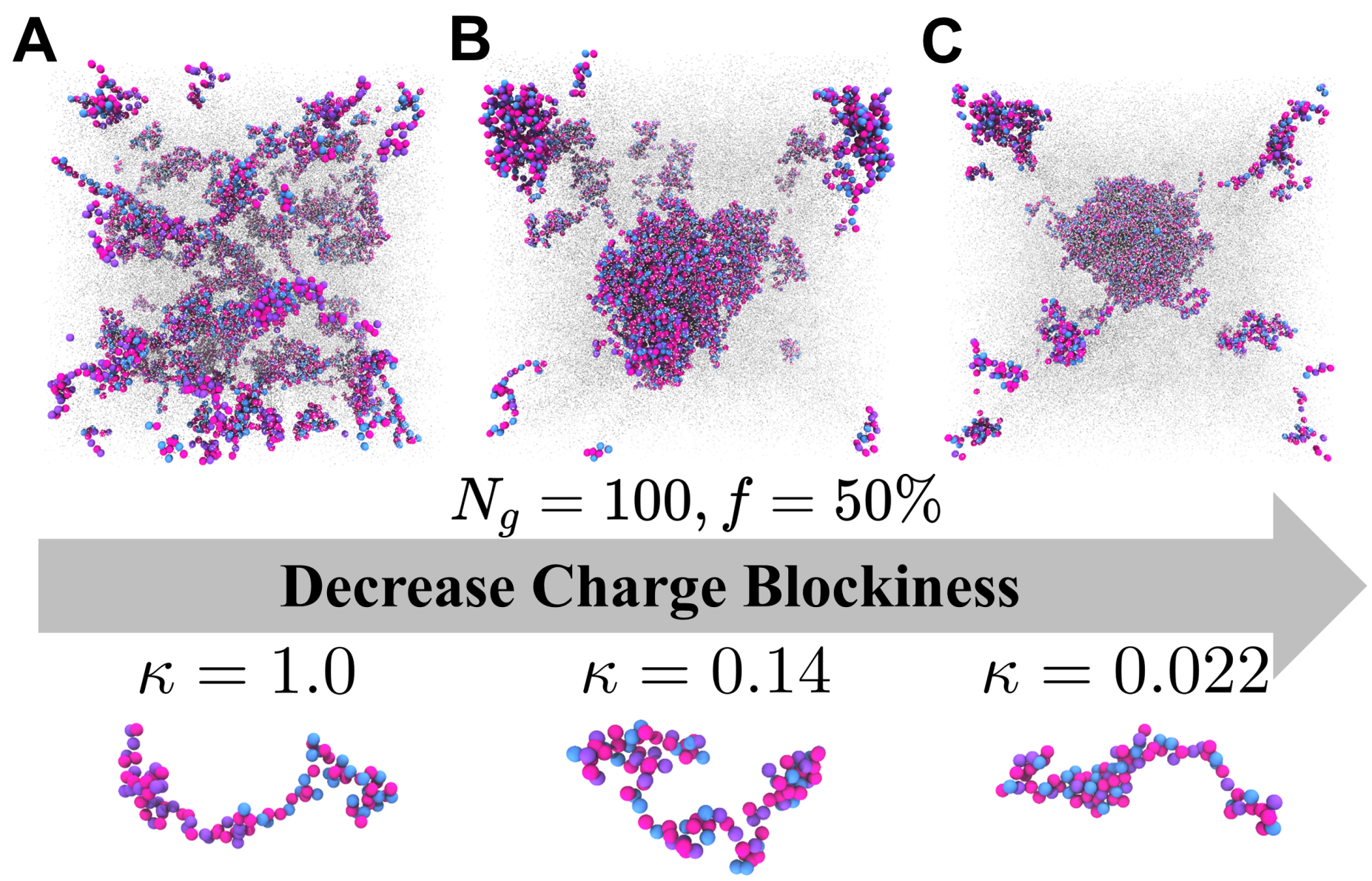

Figure S4: The behavior of 50% charged guest IDPs in the coacervate matrix ($N_h = 30$). Gray beads represent monomers of the background coacervate matrix; magenta, blue, and purple represent the neutral, negatively charged, and positively charged monomers, respectively. The water and counterions are omitted for clarity.

S7 The PMF of guests in different solvents and the decomposition of the PMF in coacervate

To further demonstrate that the electrostatic exclusion effect comes from charge-charge correlation within the coacervate matrix, we compared the effective two-chain interaction between neutral guests with $N_g = 50$ in three different solvents: the original coacervate with $N_h = 30$, the ionic solution, and semi-dilute polyelectrolyte solutions. The latter two referenced systems are prepared by removing all the polymer connectivity, and the polymer connectivity between polyanions inside the coacervate with $N_h = 30$, respectively. The result shows that the guests are repulsive to each other in both the ionic and semi-dilute polyelectrolyte solutions, while they are attractive in the coacervate (Figure S5(A)). This difference further demonstrates that the electrostatic exclusion effect arises from the inherent electrostatic correlation of the coacervate phase.

In addition, we decompose the free energy ΔF into the electrostatic energy, the excluded volume energy U_{ex}, and polymer bond energy U_{bond} contribution. Figure S5(B) shows that the most significant energy change underlying guest aggregation is the electrostatic interaction contribution, while both the excluded volume and bond interactions exhibit negligible variation (oscillating around $0k_BT$).

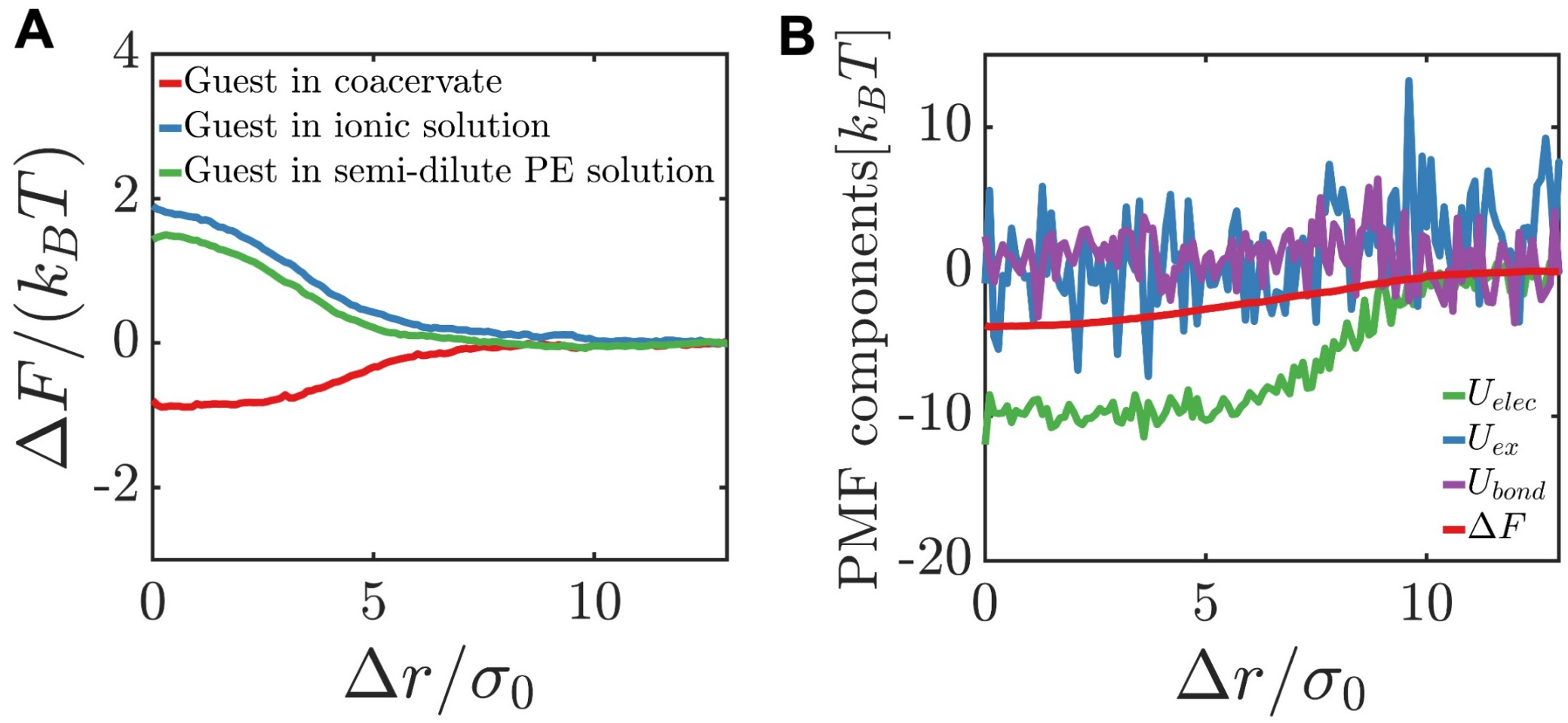

Figure S5: **A** Comparison of the effective guest-guest interaction ($N_g = 50$) within the ionic and semi-dilute polyelectrolyte solution versus the coacervate phase ($N_h = 30$). **B** Decomposition of the energy components alongside PMF of neutral guest polymer ($N_g = 100$) within the coacervate matrix ($N_h = 30$).

S8 Compression of a Single Chain in the Coacervate Phase

Electrostatic exclusion effect also significantly influences the single chain morphology, which is otherwise predicted to be as random coil in the absence of difference in excluded volume interaction between guest and host polymers [6–9]. To highlight the electrostatic effect, we calculate the end-to-end distance of a single neutral guest chain, normalized by its ideal chain conformation ($N_g^{1/2}b$). Figure S6 shows that, unless for very short chains ($N_g < 30$), the guest chain is compressed within the coacervate phase, and the compression effect enhances with increasing chain length.

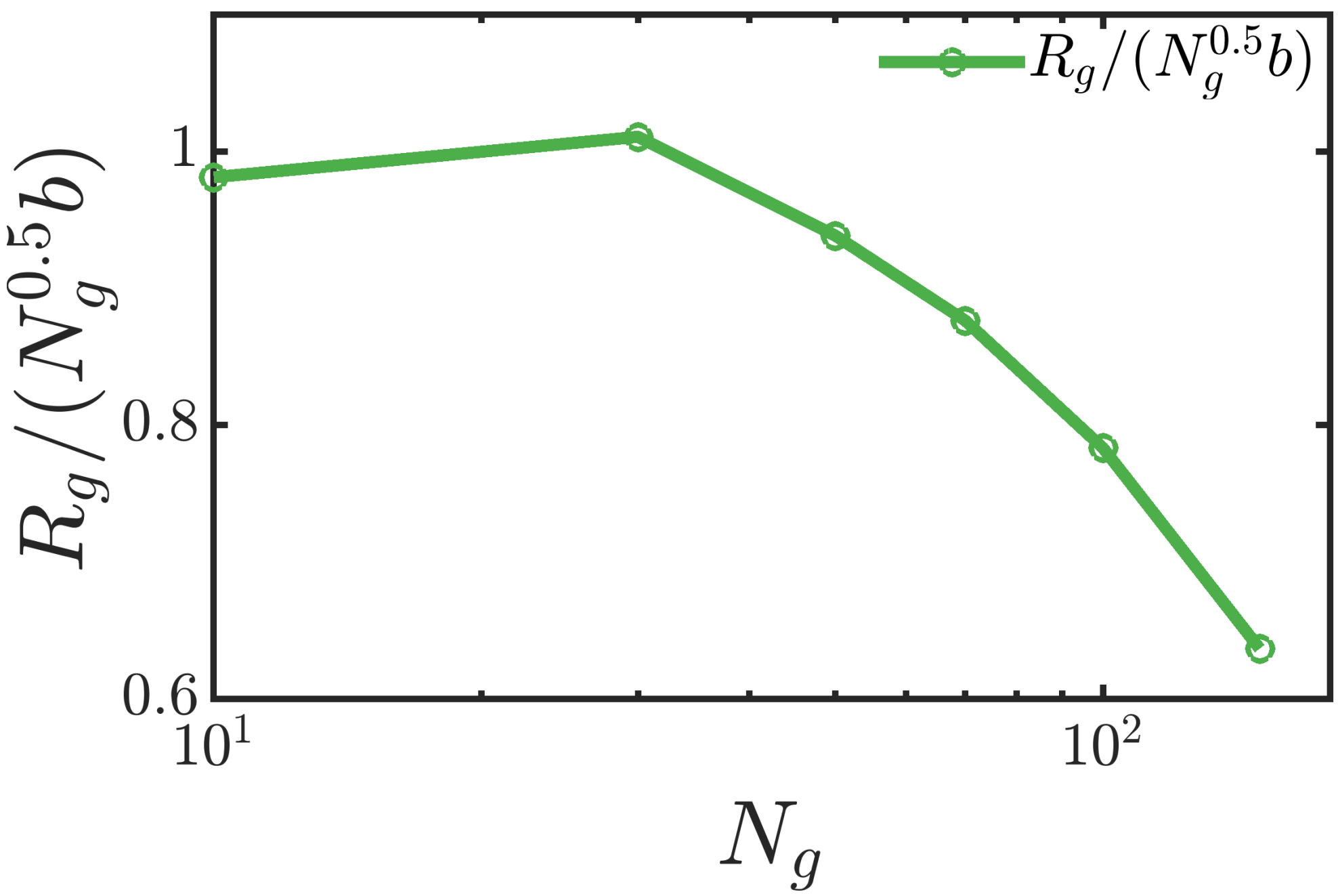

Figure S6: The end-to-end distance of a single neutral guest chain (R_g) with different chain length N_g within the coacervate phase ($N_h = 30$). Data are normalized by the ideal chain prediction $N_g^{1/2}b$, where b is the bond length.

S9 Experimental Methods

Materials

Poly(acrylic acid, sodium salt) (PA, 15000 g mol˘1), poly(allylamine) (PAH, 17000 g mol˘1), dimethyl sulfoxide (DMSO), and the fluorescent dyes fluorescein isothiocyanate and rhodamine B isothiocyanate were purchased from Sigma-Aldrich (USA). HEPES buffer (≥ 99%) was obtained in powder form from Fisher Scientific. Hen egg white lysozyme (HEWL) (≥ 95%) was supplied by Hampton Research as a lyophilized powder. For all experiments, Milli-Q water (Millipore Ltd., USA) with a resistivity of 18.2 MΩ · cm at 25 °C was used.

Poly(D, L-lysine trifluoroacetate) and poly(D, L-glutamate sodium salt), both with a polymerization degree of N = 48, were produced in-house using solid-phase peptide synthesis using a Liberty Blue automated microwave peptide synthesizer from CEM Ltd. and characterized using a Bruker UltrafleXtreme matrix-assisted laser desorption/ionization time of flight mass spectrometer (MALDI-TOF), as described previously [10]. Amino acids with alternating chirality were used to prevent backbone hydrogen bonding upon complexation [11–13].

Methods

Fluorescent Labeling Proteins and peptides were labeled by reacting primary amines with fluorescein or rhodamine isothiocyanate. A dye solution in DMSO (1 mg mL^{-1}, 50 μL) was added to the biomolecule solution (10 mg mL^{-1}, 2.5 mL in 100 mM carbonate buffer, pH 8.5) and stirred overnight at room temperature. The mixture was dialyzed (MWCO 3.5 kDa) against Milli-Q water for 5 days, lyophilized, and stored at -20 °C. Labeled biomolecules were used at 0.01-0.1 wt% relative to unlabeled polymer, depending on fluorescence intensity.

Confocal Microscopy Complex coacervates were prepared by mixing Milli-Q water, 20 mM HEPES buffer (to ensure a final pH of 7.0 ± 0.2), and oppositely charged polymers at a 1:1 charge ratio, yielding

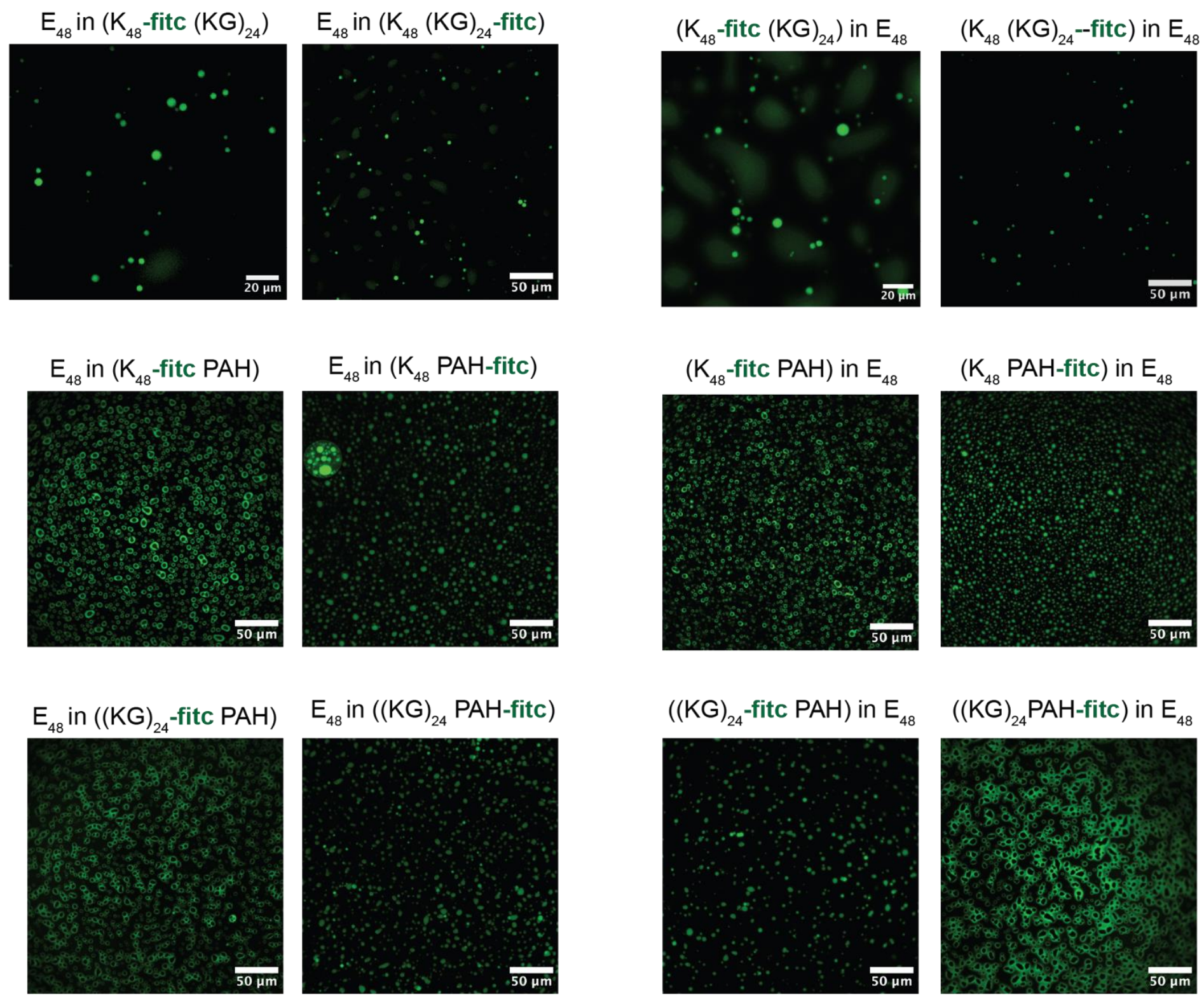

Figure S7: Influence of component addition order on the morphology and partitioning of multicomponent complex coacervates. Confocal fluorescence micrographs of coacervate droplets formed by mixing the polyanion E_{48} with binary combinations of polycations. The rows indicate the specific polycation pairs utilized: K_{48} and $(KG)_{24}$ (top row), K_{48} and PAH (middle row), and $(KG)_{24}$ and PAH (bottom row). Within each condition, a single polycation species was fluorescently labeled with FITC to visualize its spatial distribution and partitioning behavior.

a final concentration of 10 mM on a charge basis. Samples were vortexed after each addition. Droplet suspensions (20–30 μL) were deposited onto glass coverslips and imaged using a Zeiss LSM780-NLO confocal microscope equipped with a 20 × dry objective. Excitation was performed with 488 nm (fluorescein) and 561 nm (rhodamine) laser lines using a main beam splitter (MBS 488/561). Emission was collected with a 495–550 nm filter for fluorescein and a 565–705 nm filter for rhodamine.

Confocal images were analyzed using ImageJ. To determine the partitioning of the polycations, the positively charged species (PAH, K_{48}, and $(KG)_{24}$) were fluorescently labeled with FITC. Samples comprising three charged species (E_{48} and two distinct polycations) were prepared, wherein only one polycation was labeled per experiment (results are presented in Figure S7). Partitioning coefficients were calculated as the ratio of fluorescence intensity within the coacervate droplet to that in the surrounding supernatant (I_{coa}/I_{sup}), as well as the partition between the coacervate phases for the multi-phase systems (I_{inner}/I_{outer}). An analogous calculation was employed to quantify partitioning across the distinct phases of the multiphase complex considering the partition of FITC in the outer and inner complex coacervate compared to the supernatant phase.

To evaluate potential kinetic path-dependency, the sequence of component addition was varied. In the first two columns of Fig. S8B,C, the polyanion E_{48} was introduced into a pre-mixed solution of the two polycations. In the latter two columns, the pre-mixed polycation solution was added to the polyanion E_{48}. Two-sample t-tests were utilized to compare the partitioning coefficients between the two mixing sequences. For most systems, no significant difference was observed ($p > 0.05$), indicating that the final

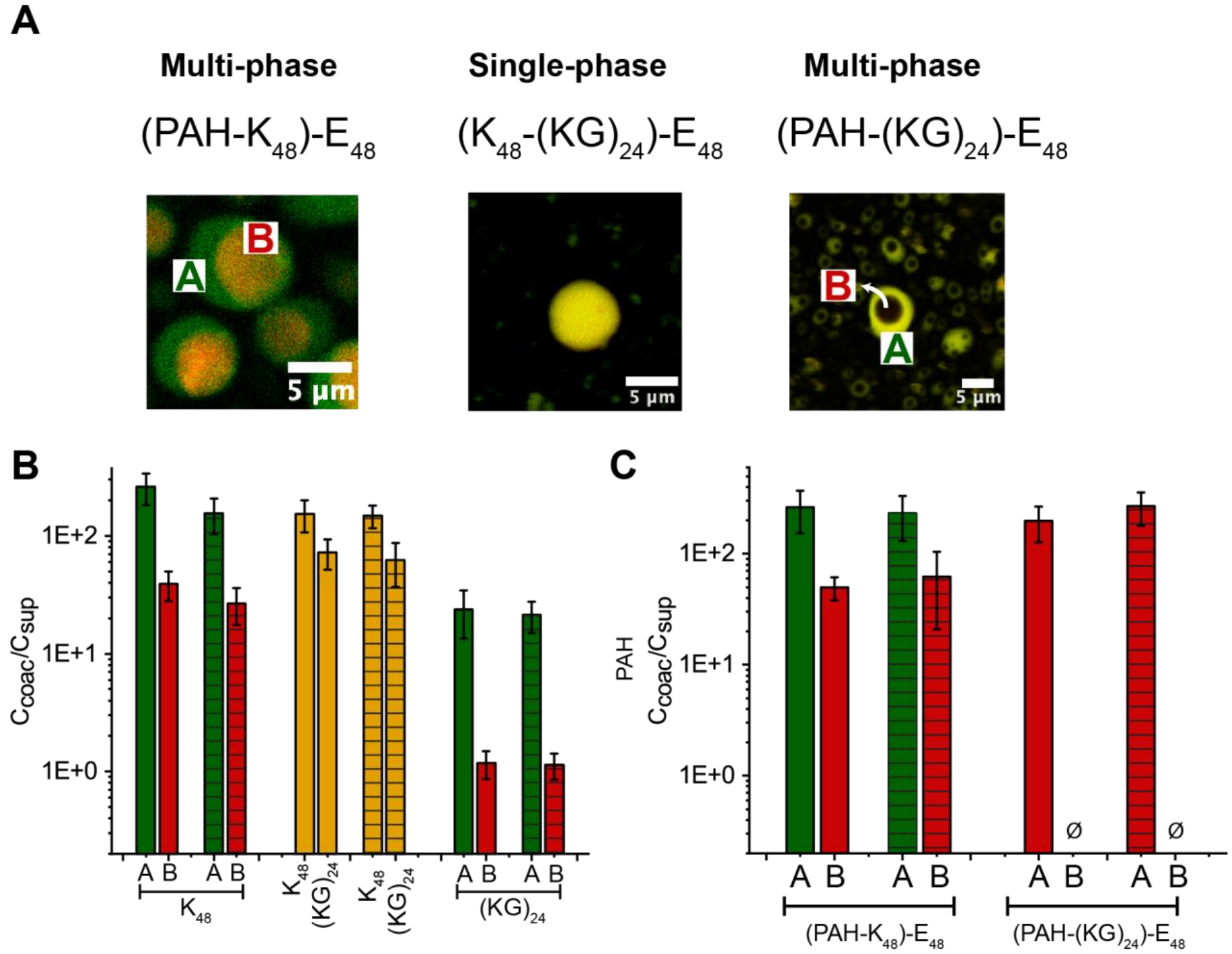

Figure S8: Phase morphology and quantitative partitioning in multicomponent coacervates. **A** Confocal micrographs of multiphase (outer green coacervate A, inner red coacervate B) and single-phase coacervates formed by mixing the polyanion E_{48} with various polycation pairs. **B,C** Partition coefficients (C_{coac}/C_{sup}) of K_{48} and $(KG)_{24}$ **B**, and PAH **C** across the distinct coacervate phases. The $\emptyset$ symbol indicates the species is completely excluded, meaning the signal between the coacervate phase and the supernatant could not be differentiated. Error bars indicate standard deviation.

state is unaffected by the order of addition. The single exception was the partitioning of K_{48} in the (PAH-K_{48})-E_{48} system, which exhibited a statistically significant difference ($p = 0.03$). Despite this quantitative difference in partitioning, the qualitative macroscopic spatial distribution of the labeled species remained consistent regardless of the mixing sequence, verifying that the overall coacervate partitioning (Fig. S8) approximates thermodynamic equilibrium.

Isothermal Titration Calorimetry (ITC) ITC measurements were carried out in triplicate on a PEAQ-ITC microcalorimeter (Malvern, USA) with a 200 μL sample cell at 298.15 $\pm$ 0.01 K. Prior to use, the instrument was cleaned, and all solutions were degassed. In a typical run, 39 injections of the polyanion solution (20 mM) were titrated into 20 mM HEPES buffer with 2 mM polycation. The initial 0.5 μL injection was discarded, while the subsequent 38 injections (1 μL each) were delivered at 600 s intervals with stirring at 500 rpm. The heat of dilution for the injection of the polyanion into 20 mM HEPES buffer was similarly measured.

Heats of dilution were subtracted from raw data, and enthalpograms were fit using a two-step coacervation model: (i) ion-pairing, described by a binding model with identical and independent sites, and (ii) phase separation leading to coacervate formation, according to previous work [10, 14–16]. From the first step, the binding constant (K_a) was determined, with errors reported as the standard deviation from fits of the three replicate experiments.

S10 Interpret electrostatic exclusion using the electrostatic blob argument

The electrostatic exclusion effect arises from the unique microscopic charge-charge correlation within the complex coacervates, where the host polyelectrolytes can be described by a sequence of electrostatic "blobs" of size ξ, with each blob preferentially surrounded by its oppositely charged counterpart[17–19] (Fig. S9). This structural correlation between oppositely charged electrostatic blobs has been confirmed by recent scattering experiments and theory [20]. Although the blob concept should only be rigorously valid for weakly charged systems [21], the qualitative picture applies to our systems in explaining the origin of electrostatic exclusion.

To quantify the characteristic length scale of the charge–charge correlations within the coacervate matrix, we first calculate the radial distribution function (RDF) between polycations and polyanions, and then apply the Fast Fourier Transform (FFT) to obtain the positive–negative charge structure factor, $G_{+-}(q)$. The first peak of $G_{+-}(q)$, denoted as q^*, corresponds to the characteristic wavevector of the polycation–polyanion correlation and defines the electrostatic blob size via $\xi = 2\pi/q^*$. In our simulated coacervate matrix with host chain length $N_h = 30$, the electrostatic blob size corresponds to a physical length of $\xi = 2.7\sigma_0 = 1.73$ nm (Fig. S9B).

The encapsulation of neutral guest polymers with chain length $N_g \geq 30$ (corresponding to a radius of gyration of around 1.6 nm) will disrupt the nanoscale (1.73 nm) charge–charge correlations between neighboring oppositely charged blobs (Fig. S9A). As a result, the guest polymers are excluded from the coacervate phase. This blob-level picture provides a nanoscale interpretation of the electrostatic exclusion.

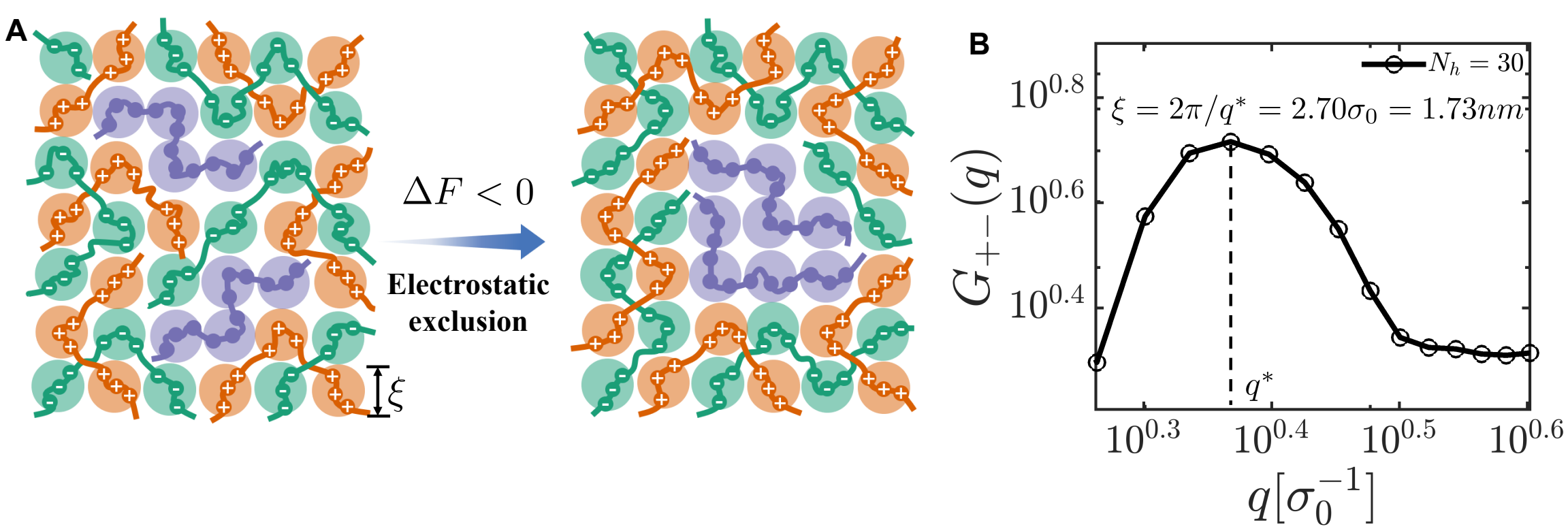

Figure S9: Interpret electrostatic exclusion using the electrostatic blob argument from polymer physics. **A**, Schematic illustrations of electrostatic correlation of oppositely charged blobs with size ξ within the coacervate matrix, which gives rise to favorable free energy changes (ΔF) upon guest aggregation. Polyanion blobs (green) are mostly surrounded by polycation blobs (orange), where guest polymers (purple blobs) can create interfaces with the charged blobs. **B**, Electrostatic blob length $\xi = 2.70\sigma_0 = 1.73$ nm calculated from the Fast Fourier Transform (FFT) of the radial distribution functions between the polycations and polyanions.

References

(1) Ye, B. B.; Chen, S.; Wang, Z.-G. GCMe: Efficient implementation of the Gaussian core model with smeared electrostatic interactions for molecular dynamics simulations of soft matter systems. *Journal of Chemical Theory and Computation* **2024**, *20*, 6870–6880.

(2) Plimpton, S. Fast parallel algorithms for short-range molecular dynamics. *Journal of Computational Physics* **1995**, *117*, 1–19.

(3) Das, R. K.; Pappu, R. V. Conformations of intrinsically disordered proteins are influenced by linear sequence distributions of oppositely charged residues. *Proceedings of the National Academy of Sciences* **2013**, *110*, 13392–13397.

(4) Darve, E.; Rodríguez-Gómez, D.; Pohorille, A. Adaptive biasing force method for scalar and vector free energy calculations. *The Journal of Chemical Physics* **2008**, *128*.

(5) Comer, J.; Gumbart, J. C.; Hénin, J.; Lelièvre, T.; Pohorille, A.; Chipot, C. The adaptive biasing force method: Everything you always wanted to know but were afraid to ask. *The Journal of Physical Chemistry B* **2015**, *119*, 1129–1151.

(6) Rubinstein, M.; Colby, R. H. *Polymer physics*; Oxford University Press: 2003.

(7) De Gennes, P.-G. *Scaling concepts in polymer physics*; Cornell University Press: 1979.

(8) Doi, M.; Edwards, S. F.; Edwards, S. F. *The theory of polymer dynamics*; Oxford University Press: 1988; Vol. 73.

(9) Xu, Y.; Wang, Z.-G. Coil-to-globule transition in polymeric solvents. *Macromolecules* **2021**, *54*, 10984–10993.

(10) Chang, L.-W.; Lytle, T. K.; Radhakrishna, M.; Madinya, J. J.; Vélez, J.; Sing, C. E.; Perry, S. L. Sequence and entropy-based control of complex coacervates. *Nature Communications* **2017**, *8*, 1273.

(11) Hoffmann, K.; Perry, S.; Leon, L.; Priftis, D.; Tirrell, M.; De Pablo, J. A molecular view of the role of chirality in charge-driven polypeptide complexation. *Soft Matter* **2015**, *11*, 1525–1538.

(12) Perry, S. L.; Leon, L.; Hoffmann, K. Q.; Kade, M. J.; Priftis, D.; Black, K. A.; Wong, D.; Klein, R. A.; Pierce III, C. F.; Margossian, K. O., et al. Chirality-selected phase behaviour in ionic polypeptide complexes. *Nature Communications* **2015**, *6*, 6052.

(13) Pacalin, N. M.; Leon, L.; Tirrell, M. Directing the phase behavior of polyelectrolyte complexes using chiral patterned peptides. *The European Physical Journal Special Topics* **2016**, *225*, 1805–1815.

(14) Priftis, D.; Megley, K.; Laugel, N.; Tirrell, M. Complex coacervation of poly (ethylene-imine)/polypeptide aqueous solutions: Thermodynamic and rheological characterization. *Journal of Colloid and Interface Science* **2013**, *398*, 39–50.

(15) Priftis, D.; Laugel, N.; Tirrell, M. Thermodynamic characterization of polypeptide complex coacervation. *Langmuir* **2012**, *28*, 15947–15957.

(16) Vitorazi, L.; Berret, J.-F.; Loh, W. Self-assembly of complex salts of cationic surfactants and anionic–neutral block copolymers. Dispersions with liquid-crystalline internal structure. *Langmuir* **2013**, *29*, 14024–14033.

(17) Shusharina, N. P.; Zhulina, E. B.; Dobrynin, A. V.; Rubinstein, M. Scaling theory of diblock polyampholyte solutions. *Macromolecules* **2005**, *38*, 8870–8881.

(18) Rumyantsev, A. M.; Zhulina, E. B.; Borisov, O. V. Complex coacervate of weakly charged polyelectrolytes: Diagram of states. *Macromolecules* **2018**, *51*, 3788–3801.

(19) Rubinstein, M.; Liao, Q.; Panyukov, S. Structure of liquid coacervates formed by oppositely charged polyelectrolytes. *Macromolecules* **2018**, *51*, 9572–9588.

(20) Fang, Y. N.; Rumyantsev, A. M.; Neitzel, A. E.; Liang, H.; Heller, W. T.; Nealey, P. F.; Tirrell, M. V.; de Pablo, J. J. Scattering evidence of positional charge correlations in polyelectrolyte complexes. *Proceedings of the National Academy of Sciences* **2023**, *120*, e2302151120.

(21) Rumyantsev, A. M.; Johner, A. Electrostatically Stabilized Microstructures: From Clusters to Necklaces to Bulk Microphases. *ACS Macro Letters* **2025**, *14*, 472–483.
